# The zinc finger protein DCM1 is required for male meiotic cytokinesis by preserving callose in rice

**DOI:** 10.1371/journal.pgen.1007769

**Published:** 2018-11-12

**Authors:** Chao Zhang, Yi Shen, Ding Tang, Wenqing Shi, Dongmei Zhang, Guijie Du, Yihua Zhou, Guohua Liang, Yafei Li, Zhukuan Cheng

**Affiliations:** 1 State Key Laboratory of Plant Genomics and Center for Plant Gene Research, Institute of Genetics and Developmental Biology, Chinese Academy of Sciences, Beijing, China; 2 University of Chinese Academy of Sciences, Beijing, China; 3 Jiangsu Co-Innovation Center for Modern Production Technology of Grain Crops, Yangzhou University, Yangzhou, China; Fudan University, CHINA

## Abstract

Meiotic cytokinesis influences the fertility and ploidy of gametes. However, limited information is available on the genetic control of meiotic cytokinesis in plants. Here, we identified a rice mutant with low male fertility, *defective callose in meiosis 1* (*dcm1*). The pollen grains of *dcm1* are proved to be defective in exine formation. Meiotic cytokinesis is disrupted in *dcm1*, resulting in disordered spindle orientation during meiosis II and formation of pollen grains with varied size and DNA content. We demonstrated that meiotic cytokinesis defect in *dcm1* is caused by prematurely dissolution of callosic plates. Furthermore, peripheral callose surrounding the *dcm1* pollen mother cells (PMCs) also disappeared untimely around pachytene. The DCM1 protein contains five tandem CCCH motifs and interacts with nuclear poly (A) binding proteins (PABNs) in nuclear speckles. The expression profiles of genes related to callose synthesis and degradation are significantly modified in *dcm1*. Together, we propose that DCM1 plays an essential role in male meiotic cytokinesis by preserving callose from prematurely dissolution in rice.

## Author summary

Meiosis comprises two successive cell divisions after a single S phase, generating four haploid products. Meiotic caryokinesis (nuclear division) has been extensively studied in many organisms, while mechanisms underlying meiotic cytokinesis remain elusive. Here, we identified a novel CCCH-tandem zinc finger protein DCM1 that prevent the premature dissolution of callose both around the PMCs and at the dividing site (callosic plates). Loss of the callosic plates disrupts the meiotic cytokinesis, leading to the random distribution of spindles during meiosis II and aberrant meiotic products. DCM1 interacts with the two rice poly (A) binding proteins, independently of the conserved CCCH domain. Moreover, DCM1 coordinates the expression profiles of genes related to callose synthesis and degradation. We suspect monocots and dicots may adopt distinct meiotic cytokinesis patterns during male gamete generation.

## Introduction

Cytokinesis is the process by which the two daughter nuclei resulting from nuclear division are physically separated by the establishment of a cell plate and/or cell wall. In animal and yeast dividing cells, an actomyosin ring contracts centripetally to separate the daughter cells [1]. In higher plants, however, cytokinesis involves the formation of a cell plate through the fusion of vesicles at its centrifugally expanding periphery [2, 3]. Meiosis is a specialized type of cell division consisting of one round of DNA replication and two rounds of nuclear division [4]. In plant male meiosis, two different types of cell plate formation, namely successive and simultaneous cytokinesis, are documented. Each caryokinesis is directly followed by a cytokinetic event in successive cytokinesis (typically in monocots), while the simultaneous cytokinesis occurs only when both nuclear division are finalized (typically in dicots) [5].

Callose, a β-1, 3-glucan polymer with β-1, 6-branches, plays important roles in response to biotic and abiotic stresses, as well as in a variety of developmental processes, especially in cell plate formation and reproductive development in plants [6, 7]. During mitosis, callose is deposited at the cell plate during the tubular network stage and is later replaced by cellulose [8]. Mutation of *MASSUE/AtGSL8 *in *Arabidopsis*, which encodes a putative callose synthase, leads to seedling lethality and a striking cytokinesis-defective phenotype [9, 10]. Moreover, *enlarged tetrad 2*, which harbors a splice site mutation of *AtGSL8*, undergoes premeiotic endomitosis due to cytokinetic defects in flowers [11]. These findings indicate that callose is required for plant mitotic cytokinesis.

For pollen mother cells (PMCs), callose is placed both at the division site as well as at the outer cell wall. The peripheral callose may prevent PMCs fusion and cohesion, and appear to participate in the formation of the primexine by providing a mold for pollen exine construction during microsporogenesis [12]. Callose synthase 5 (CalS5), also known as glucan synthase-like 2 (AtGSL2), is responsible for the synthesis of callose deposited at the primary cell wall of PMCs, tetrads and microspores, and is essential for exine formation of pollen wall in *Arabidopsis* [12, 13]. Mutation of *OsGSL5*, the rice homolog of *AtGSL2*, also results in defective callose deposition and abnormal pollen exine structure formation [14]. *AtGSL1* and *AtGSL5*, two closely related genes in *Arabidopsis*, are required for synthesis of the interstitial callose that normally separates microspores. The enlarged pollen grains and multi-nucleate microspores in *gsl1-1*/+ *gsl5-2*/*gsl5-3* suggest that callose is required for meiotic cytokinesis [15]. In *Arabidopsis mpk4*, which has a male-specific meiotic cytokinesis defect, PMCs fail to form normal intersporal callose walls after meiosis, and thus cannot complete meiotic cytokinesis [16]. Moreover, TETRASPORE/STUD, a kinesin required for male meiotic cytokinesis in *Arabidopsis *[17, 18], was reported to function upstream of MPK4 in a putative cascade pathway.

CCCH zinc finger proteins are characterized by a zinc finger motif consisting of three cysteines and one histidine coordinated by a zinc cation and have been identified in *Arabidopsis*, rice [19] and other plant species [20–22]. Recent studies have revealed that CCCH proteins participate in the regulation of many developmental processes and environmental responses [23, 24]. Although the molecular functions have not been fully characterized, multiple lines of evidence suggest that proteins with CCCH motifs can regulate gene expression through modulation of RNA metabolism. For example, OsTZF1 binds to U-rich sequences in the 3’UTR of two potential target mRNAs *in vitro* [25], and AtTZF1 can trigger the degradation of AU-rich elements (ARE)-containing mRNA *in vivo* [26].

Here, we report a novel protein, DCM1 (Defective Callose in Meiosis 1), is required for meiotic cytokinesis in rice. The meiotic cytokinesis defect is caused by the premature dissolution of callosic plates during both telophase I and telophase II. DCM1 interacts with two nuclear poly (A) binding proteins, which are members of the polyadenylation factor family, independently of the conserved tandem CCCH domain. Our study provides insight into the mechanism underlying meiotic cytokinesis in monocots.

## Results

### Identification of a mutant with dramatically reduced fertility

**Fig 1:**
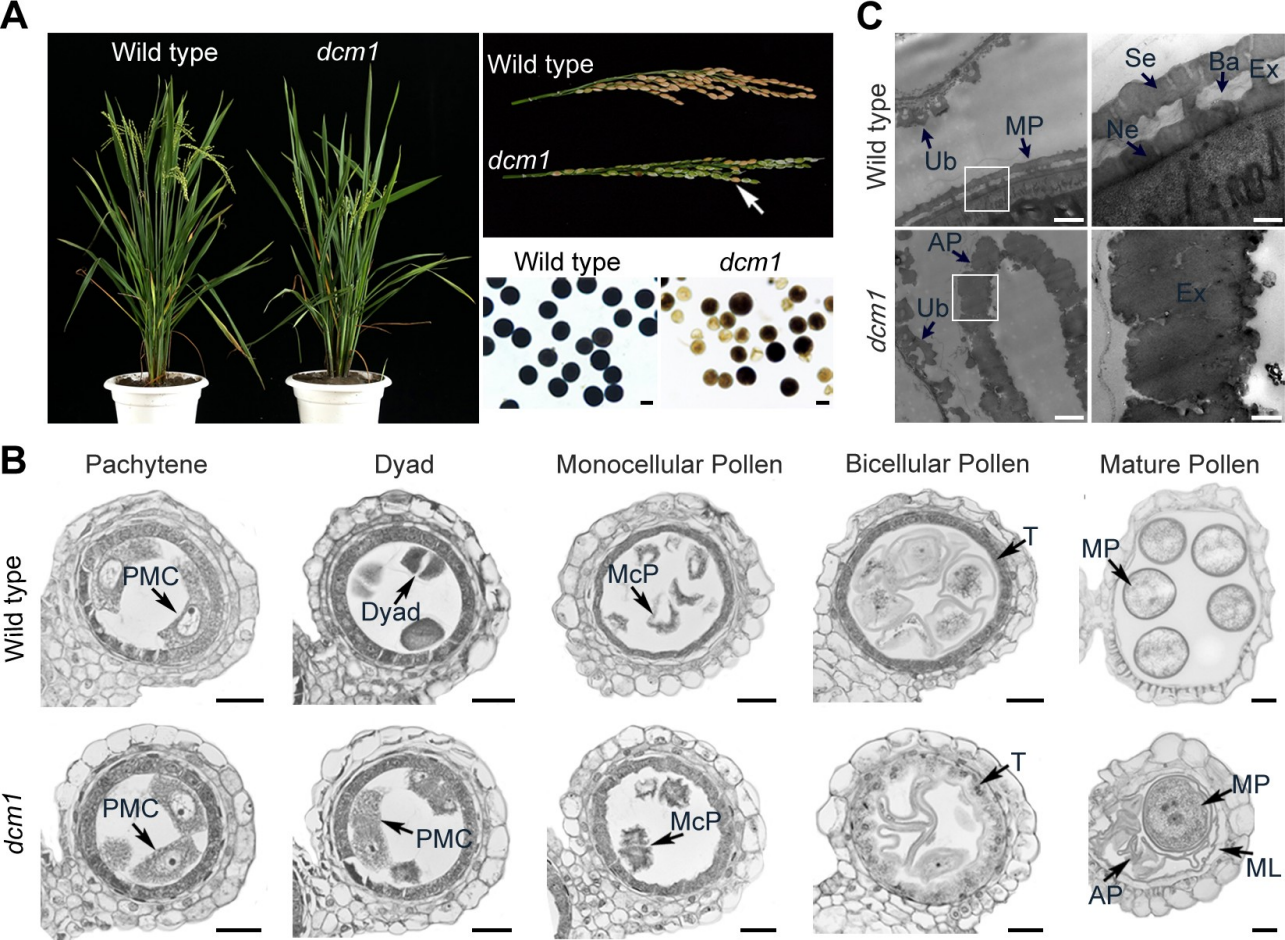
*dcm1* is a mutant with low male fertility. (A) Phenotypic comparison of plant (Left), panicle (Upper Right, a few seeds of *dcm1* indicated by an arrow) and pollen grains (Lower Right) of wild type and *dcm1*. Bars = 20μm. (B) Semi-thin sections of anthers from wild type and *dcm1* at different developmental stages. Bars = 20μm. (C) Transmission electron micrographs of the anthers from both wild type and *dcm1*. Bars *=* 1μm in the left column and 200nm in the right column. AP, aborted pollen; Ba, bacula; Ex, exine; ML, middle layer; MP, mature pollen; McP, monocellular pollen; Ne, nexine; PMC, pollen mother cell; Se, sexine; T, tapetum; Ub, Ubisch body.

We isolated the *defective callose in meiosis 1* (*dcm1*) mutant in a screen of our rice mutant libraries with reduced fertility. The *dcm1* mutant exhibited no defects in vegetative growth but was nearly sterile with panicles occasionally bearing a few seeds (Fig 1A). When stained with I_2_-KI, most pollen grains (85.1%, n = 766) were less stained or even shrunken compared with wild-type pollens, indicating the dramatically reduced viability of male gametes. The viability of *dcm1* female gametes was evaluated by pollinating its flowers with wild-type pollen grains. The resulting normal seed setting rate indicated that female fertility was not affected in *dcm1* (S1 Table).

To investigate the cellular defects of *dcm1*, we examined transverse sections of both wild-type and mutant anthers at different developmental stages (Fig 1B). During early meiosis, four layers of anther somatic cells (epidermis, endothecium, middle layer and tapetum) enclosed the anther locule, where PMCs contacted with the tapetal layer. After meiosis, the PMCs gave rise to microspores. Among these stages, no significant differences were observed between wild-type and *dcm1* anthers. During the bicellular pollen stage, wild-type pollens were vacuolated with an increased volume and tapetal cells were deeply stained with toluidine blue. By contrast, tapetal cells of *dcm1* were larger than normal with reduced staining and started to degenerate at this stage. During the mature pollen stage, all wild-type pollen grains were round and the inner layers of the anther degenerated. However, besides round pollen grains, shrunken empty pollen grains (75%, n = 56) were observed in *dcm1*. In addition, the middle layer, which was not visible in mature wild-type anther, was swollen in the mutant.

To obtain a more detailed understanding of the abnormalities of the *dcm1* pollen grains, mature pollen was visualized using transmission electron microscopy (TEM) (Fig 1C). Ubisch bodies, specialized structures forming along the inner surface of the tapetum, seemed to be normal in *dcm1*. The wild-type pollen grains had completed pollen exine deposition and exhibited distinctive sublayers, including sexine and nexine. However, only one layer, which seemed to be uniform in component, was observed in *dcm1* pollen grains (n = 17), indicating that the formation of pollen exine was disrupted in *dcm1*.

### Abnormal meiotic products give rise to pollen grains of varied size in *dcm1*

**Fig 2:**
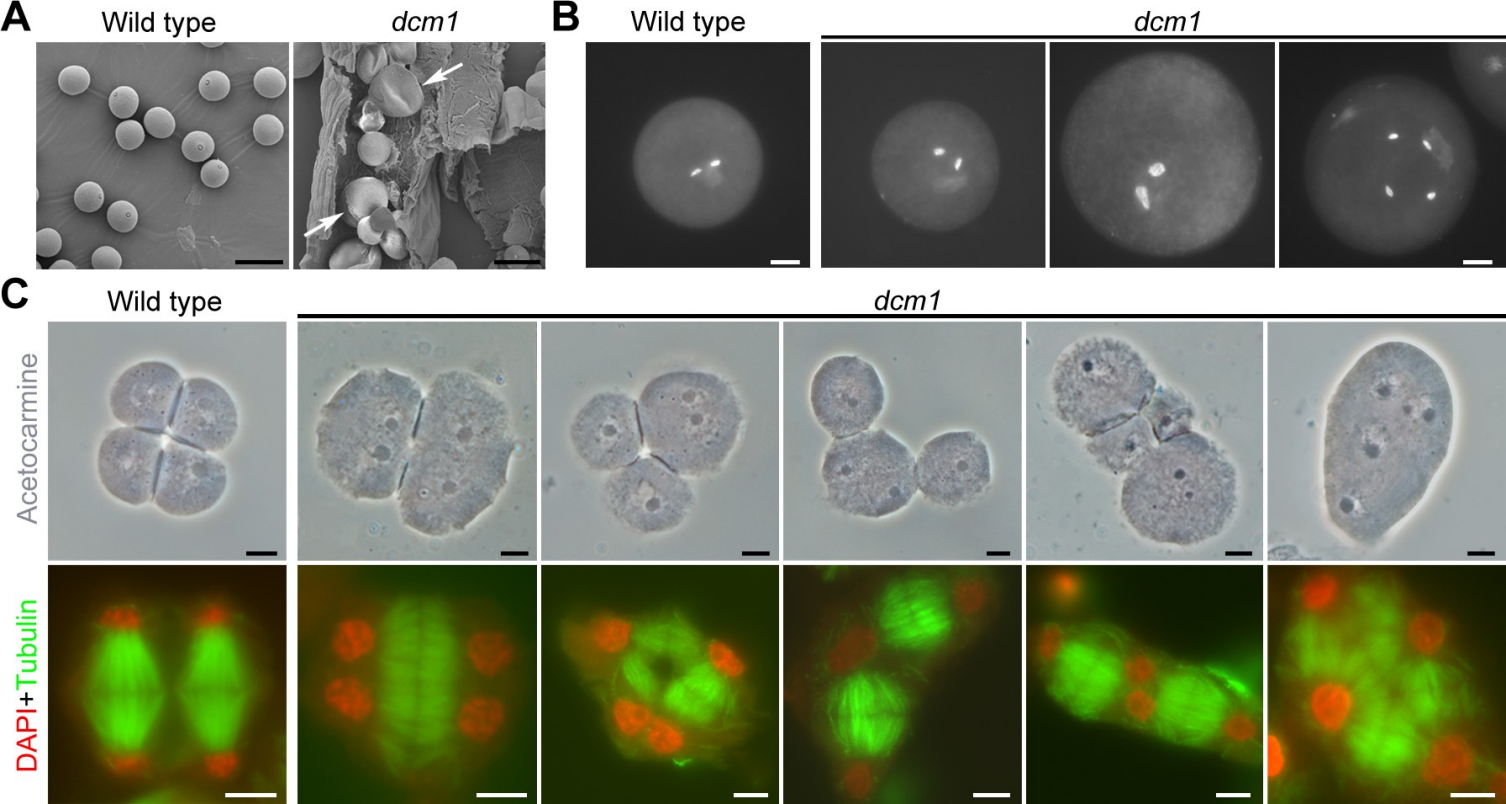
Pollen grains with non-uniform size in *dcm1*. (A) Scanning electron microscope observation of wild-type and *dcm1* pollen grains. Large pollen grains are indicated by arrows. Bars = 50μm. (B) DAPI stained pollen grains in wild-type and *dcm1*. Bars = 10μm. (C) The arrangement of meiotic products and orientation of spindles in wild type and *dcm1*. Meiotic products are stained with acetocarmine. Spindles are immunolocalized with α-tubulin antibody. Bars = 5μm.

The size of the pollen grains of *dcm1* seemed to be nonuniform (Fig 1A and Fig 1B). To confirm this, we observed the mature pollen grains of wild type and *dcm1* using scanning electron microscope (SEM). The wild-type pollen grains were round and uniform in size (n = 182). However, the pollen grains of the mutant varied in size, with some pollen grains (41.7%, n = 206) obviously larger than the wild type (Fig 2A).

As the pollen size is always correlated with its ploidy [27], the nuclear DNA of pollen grains was stained with 4’, 6-diamino-phenylindole (DAPI) (Fig 2B). In the 238 checked pollen grains of *dcm1*, 153 normal-sized *dcm1* pollen grains (64.3%) had a wild-type-like nuclei configuration and staining intensity. However, 78 enlarged pollen grains (32.8%) with more intensively stained and enlarged nuclei were observed in *dcm1*, suggesting increased gametophytic DNA content in the mutant. Doubled nuclei were also observed in seven larger pollen grains (2.9%) among the 238 checked pollen grains of *dcm1*.

PMCs give rise to a group of four haploid spores through meiosis. Each uninucleate microspore undergoes an asymmetric mitotic division to produce the vegetative cell and the generative cell. The generative nucleus completes the second mitotic division, developing into trinucleate pollen. To clarify the time when the defect of *dcm1* occurred, before or after the microspore stage, we observed the microspores of both wild type and *dcm1* (S1 Fig). Most microspores (98%, n = 200) of wild type were uniform in size, with the diameter ranging from 17 μm to 23 μm. However, the sizes of microspores in *dcm1* were variable (n = 277), ranging from 15 μm to 47 μm.

We then checked the meiotic products of wild type and *dcm1* by acetocarmine dyeing (Fig 2C). In wild-type tetrad, four microspores were held together in a tetragonal shape. However, the arrangement of meiotic products in *dcm1* varied. In 95 checked PMCs of *dcm1*, 18 PMCs (18.9%) divided into two daughter cells and each cell contained two nuclei. 43 PMCs (45.3%) divided into three daughter cells with one nucleus in each small cell and two nuclei in the big one. In addition, four daughter cells, each of which had one nucleus (14.7%), and one daughter cell with four nuclei (21.1%) were also observed in *dcm1*.

Spindle organization during telophase II was investigated by performing immunolocalization with α-tubulin antibody (Fig 2C). In wild-type PMCs, two sets of spindles were roughly parallel to each other, leading to the formation of four well separated poles at telophase II. However, most checked PMCs (n = 67) in *dcm1* had fused spindles (13.4%), tripolar spindles (59.7%) or linear spindles (7.5%). Among them, spindles in seven *dcm1* PMCs (10.4%) linked every two nuclei when they were adjacent to each other, which was reminiscent of the spindle arrangement during simultaneous meiotic cytokinesis.

### Meiotic cytokinesis is defective in *dcm1*

**Fig 3:**
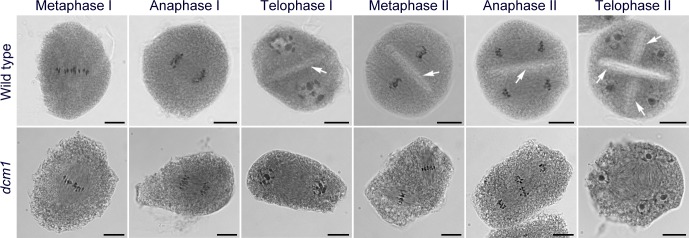
Meiotic cytokinesis is defective in *dcm1*. PMCs of wild type and *dcm1* at different developmental stages are shown. Cell plates are indicated by arrows. Bars = 10μm.

The abnormal spindle arrangements observed in *dcm1* during telophase II indicated that meiotic cytokinesis might be defective, leaving the spindle physically unseparated and free to move. As a monocot, rice undergoes successive cytokinesis during meiosis, in which the dyad is generated after meiosis I and the tetrad is formed after meiosis II. In the wild-type PMCs, homologous chromosomes segregated and were pulled to opposite poles during meiosis I (Fig 3). A cell plate was established that insulated the two chromatin groups and cytoplasm at telophase I. The cell plate persisted through metaphase II when the chromosomes aligned at the equatorial plate and anaphase II when the chromatids were pulled toward opposite poles. Two new cell plates, both perpendicular to the first one, were formed during telophase II and tetrads were then produced.

The meiotic stages from metaphase I to anaphase I in *dcm1* were roughly the same as those in wild type (Fig 3). However, no cell plate was formed in most PMCs (94.4%, n = 72) during telophase I. The two equatorial plates and spindles were not parallel to each other in *dcm1* during metaphase II. During anaphase II, the separating sister chromatids were pulled in varied orientations. Without the cell plate, meiosis II took place in a single cell. We also observed defects in the second meiotic cytokinesis in the mutant, which gave rise to microspores with four nuclei.

### Prematurely dissolution of callose occurs in *dcm1* PMCs

**Fig 4:**
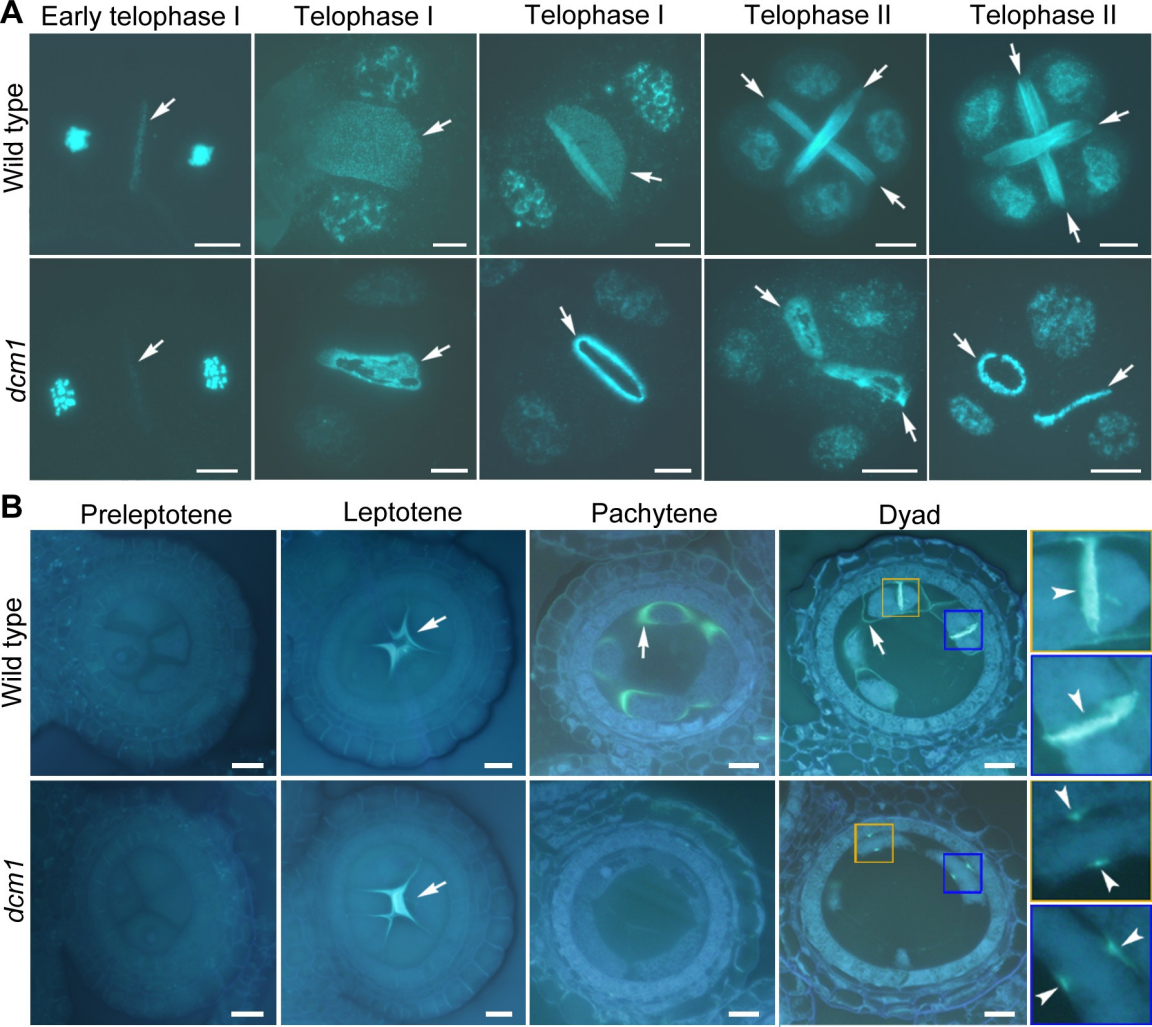
Prematurely dissolution of callosic plates and peripheral callose occur in *dcm1* PMCs. (A) Combined DAPI and aniline blue staining on wild-type and *dcm1* PMCs at different developmental stages. Callose signals are indicated by arrows. Bars = 10μm. (B) Aniline blue staining on semi-thin section of anther in wild type and *dcm1*. Peripheral callose is indicated by arrows and callosic plates are indicated by arrow heads. Callose signals at dyad are indicated in yellow and blue frames. Bars = 10μm.

Previous studies have demonstrated that cell plate formation in successive-type PMCs follows a similar pattern as somatic cytokinesis, in which callose plays an essential roles [5, 9, 10]. So, we monitored callosic plate formation in wild-type and *dcm1 *PMCs by combined DAPI and aniline blue staining. Aniline blue binds to callose and emits fluorescence under ultraviolet light. In wild-type anaphase I PMCs, callose was absent between the two separating chromosome mass. When the chromosomes reached the opposite poles during early telophase I, only weak callose signals appeared in the center of the PMCs (Fig 4A). As chromosomes decondensed into chromatin during telophase I, a callosic plate was formed. At the end of meiosis, two additional callosic plates were formed and four chromatin masses were separated.

In *dcm1*, aberrance was first observed at telophase I, which corresponds to the time when the callosic plate was formed in wild-type PMCs. Although intact callosic plates were occasionally observed in a few cells (3.6%, n = 138), incomplete callosic plates with an intact periphery and a broken inner plane were always observed (Fig 4A). During meiosis in rice, callosic plate formation occurs in an outward direction (S2 Fig) [28], indicating that the callosic plates with broken inner planes in *dcm1* were in the process of dissolution, not formation. After the complete dissolution of the inner plane, only a callose ring was observed between the chromatin groups. This callose ring, which was presented as two dot-like callose signals in anther transverse sections (88.2%, n = 51), was closely associated with the periphery of the parental PMCs (Fig 4B). Callosic plates with broken inner planes and callose rings were also observed during telophase II, accompanied by a disordered distribution of nuclei. In wild type PMCs, callosic plates persisted to the tetrad stage. Our observations suggested that prematurely dissolution of callosic plates occurred during both telophase I and telophase II in *dcm1*.

During rice meiosis, callose is deposited both at the cell plate and around the PMCs. To determine whether the callose surrounding the PMCs was also disrupted in the mutant, we compared the callose pattern in wild type and *dcm1* by staining anther transverse sections (Fig 4B). At preleptotene stage, the very beginning of meiosis, callose was detectable neither in wild type nor in *dcm1*. In wild-type PMCs, callose first appeared in the center of the locule at leptotene and extended to form an intact callose wall at pachytene. However, despite the normal callose deposition at leptotene, no callose signal around *dcm1* PMCs (n = 72) was observed at pachytene and later meiotic stages. These observations indicated that peripheral callose is also under untimely dissolution in *dcm1* PMCs.

**Fig 5:**
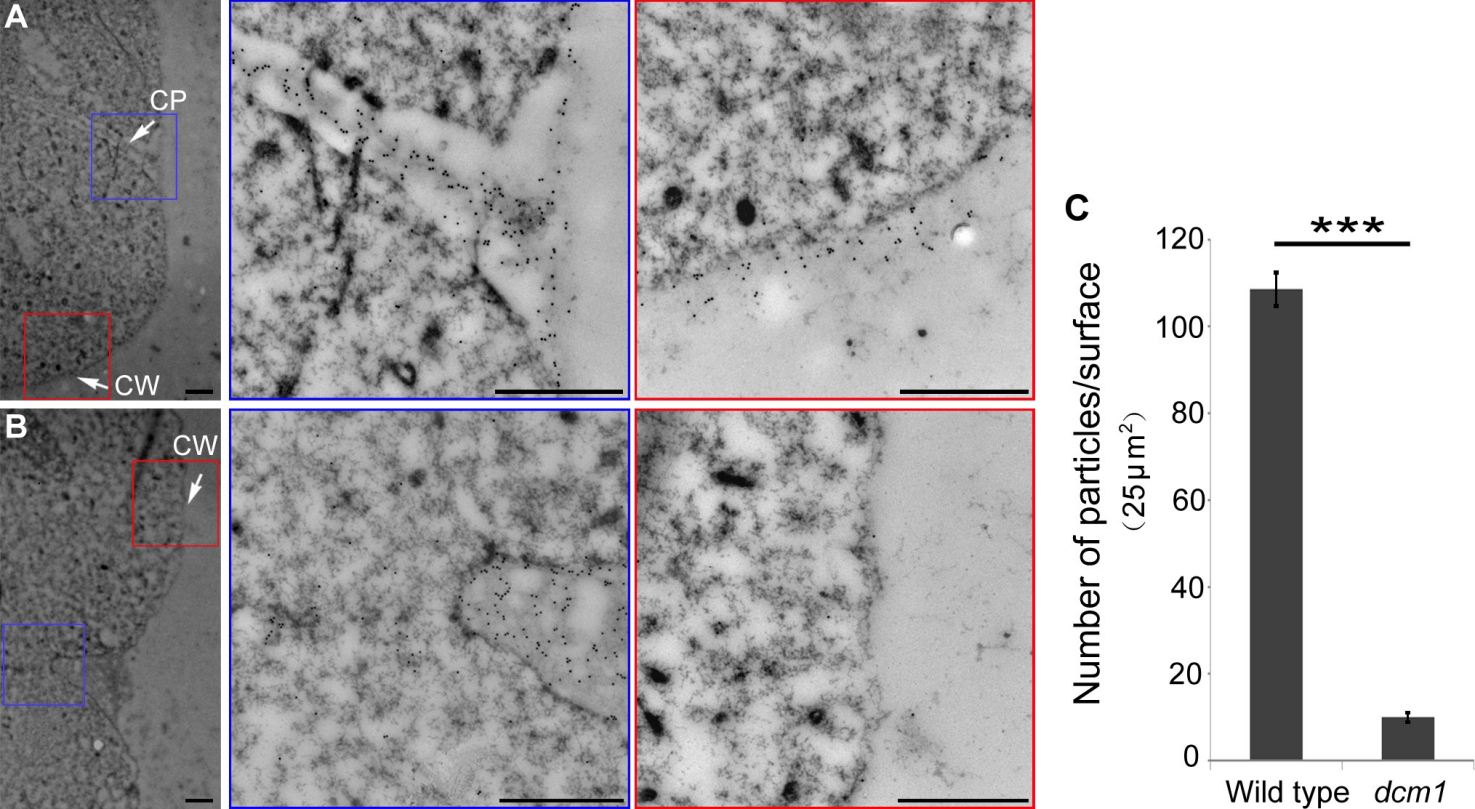
Electron micrographs showing immunogold labeling of callose in wild type and *dcm1*. (A) Dyads of the wild type. (B) Dyad-like stage in *dcm1*. CP, cell plate, enlarged in blue frames. CW, cell wall, enlarged in red frames. Bars = 1μm. (C) Quantification of immunogold labeling particles per unit area in the cell wall. Error bars represent SD (n = 50). Asterisks indicate significant differences according to Student’s *t*-test (***P<0.001).

Immunogold assay using antibody to β-1, 3-glucan showed similar callose defect in *dcm1* (Fig 5). In wild-type dyad, callose signals were observed both in cell plate and cell wall (Fig 5A). In the PMCs of *dcm1* during corresponding stage, cell plate was not formed and callose located in cell wall was almost completely lost (Fig 5B and 5C). Corresponding to the dot-like callose signals in semi-thin transverse sections (Fig 4B), immunogold labeling particles were only observed in the region of cell wall to which the hypothetical cell plate attached in *dcm1*.

### *DCM1* encodes a protein with five tandem CCCH motifs

The progenies of a *dcm1* heterozygous plant segregated for fertile and sterile phenotypes in a ratio of approximately 3:1 (154:48, χ2 = 0.165, P>0.05), indicating that the mutant phenotype was caused by a single recessive nuclear gene mutation. Through map-based cloning (S3 Fig) and next-generation sequencing, a nucleotide deletion in the first exon of *LOC4341610* was found. We knocked out this gene in wild-type plants using a CRISPR/CAS9 gene editing approach. The resulting transgenic lines displayed defective meiotic cytokinesis, mimicking the phenotype of *dcm1* (S4 Fig). This confirmed that the mutant phenotype is indeed caused by *LOC4341610* dysfunction.

**Fig 6:**
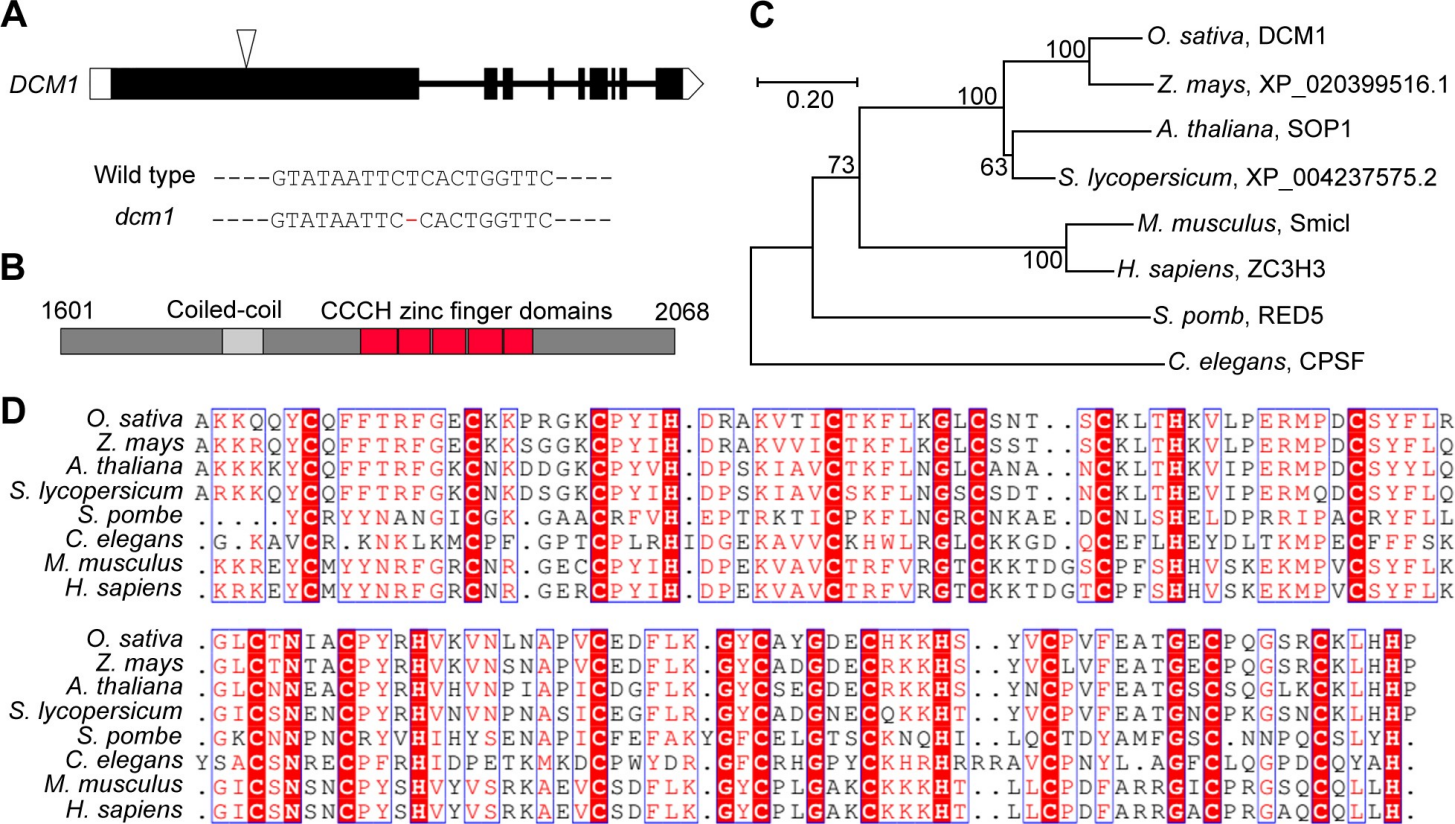
*DCM1* encodes a protein with five tandem CCCH motifs. (A) Gene structure of *DCM1*. Coding regions are shown as black boxes. 5’ and 3’ untranslated regions are shown as white boxes. Introns are shown as black lines. The triangles indicate the mutated sites in *dcm1*. Details of the mutated site are listed below. (B) Schematic representation of the conserved coiled-coil and tandem CCCH zinc finger domains in DCM1 protein. (C) Phylogenetic tree derived from the full length amino acid sequences of proteins containing the five tandem CCCH zinc finger motifs. The tree was constructed using Mega7 based on the neighbor-joining method. (D) Multiple sequence alignment of the tandem CCCH zinc finger domains from proteins used in phylogenetic tree construction.

We obtained a 6807 bp full-length cDNA of *DCM1* by performing rapid amplification of cDNA ends (RACE) (Fig 6A). The open reading frame (ORF) is 6207 bp in length, and the deduced protein contains 2068 amino acids. The DCM1 protein contains five tandem CCCH type zinc finger motifs at its C terminus (Fig 6B). Phylogenetic analysis showed that DCM1 is more closely related to its plant homologs, which constitute an isolated branch in the phylogenetic tree (Fig 6C). Multiple sequence alignments of the tandem CCCH domain from different species revealed that this domain is conserved among different kingdoms (Fig 6D).

**Fig 7:**
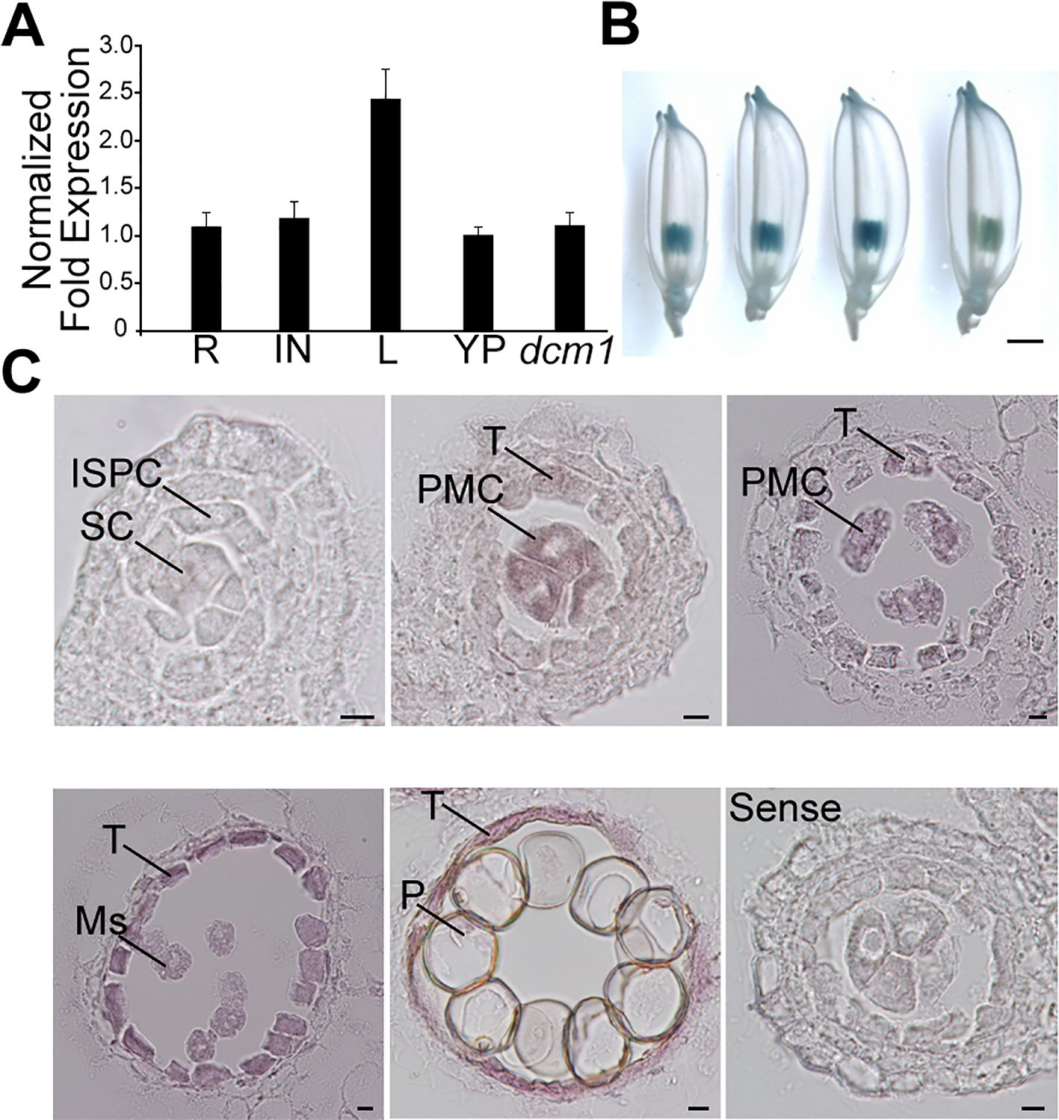
Expression pattern of the *DCM1* gene. (A) Real-time PCR analysis of *DCM1* in different tissues. *Ubiquitin* is used as endogenous control. Error bars represent SD (n = 3). R, root; IN, internode; L, leaf; YP, young panicle; *dcm1*, young panicle of *dcm1*. (B) GUS activity in the *pDCM1*::*GUS* line. The developmental stages (from left to right): leptotene, pachytene, microspore stage, pollen grain stage. Bars = 1mm. (C) RNA *in situ* analysis of *DCM1* in wild-type anthers at different developmental stages. Anther at leptotene with sense probe is shown as the negative control. Bars = 10um. ISPC, inner secondary parietal cell; Ms, microspore; P, pollen; PMC, pollen mother cell; SC, sporogenous cell; T, tapetum.

The expression profile of *DCM1* in different organs was determined by RT-PCR analysis. *DCM1* transcripts were detected in all tested organs of wild type and young panicle of *dcm1* (Fig 7A). In transgenic plants carrying the *pDCM1*::*GUS* construct, GUS signals were detectable in the anther (Fig 7B). To more precisely determine the spatial and temporal patterns of *DCM1* expression in the anthers, we performed RNA *in situ* hybridization on wild-type anther sections. No *DCM1* expression was detected in sporogenous cells in the anther during the premeiotic stage (Fig 7C). At the stage when callose began to deposit around the PMCs, *DCM1* was specifically expressed in PMCs that adhered to each other. *DCM1* expression was also observed in the tapetum during later stages.

### DCM1 interacts with nuclear poly (A) binding proteins in nuclear speckles

**Fig 8:**
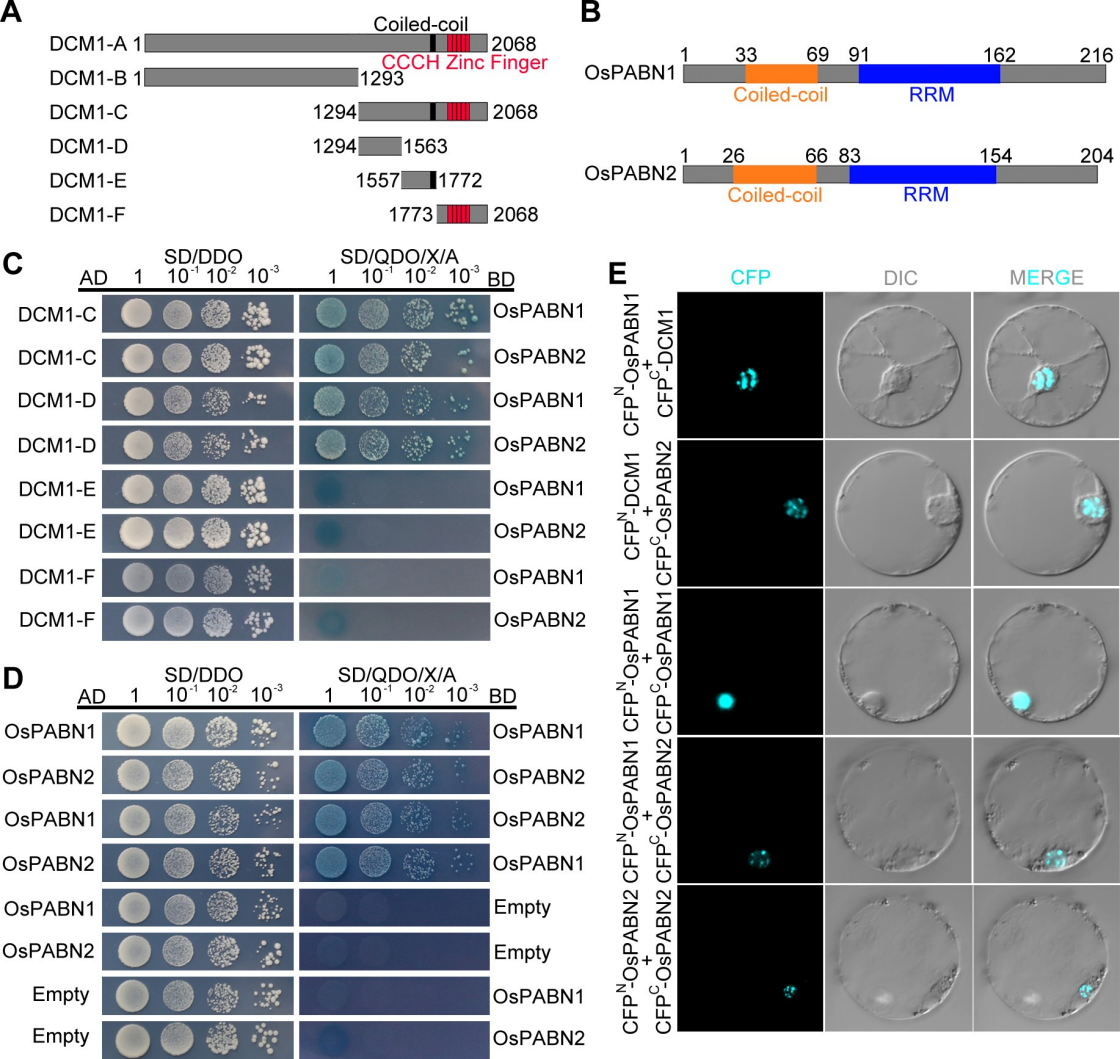
DCM1 interacts with nuclear poly (A) binding proteins in nuclear speckles. (A) Schematic diagram of full-length and truncated proteins of DCM1 used in yeast two-hybrid assays, the coiled-coil domain and tandem CCCH domain are annotated. (B) Schematic representation of OsPABN1 and OsPABN2. The conserved domains are documented. (C) DCM1 interacts with OsPABN1 and OsPABN2 in yeast two-hybrid assays, independently of the conserved tandem CCCH domain. (D) OsPABNs interacts with themselves and each other in yeast two-hybrid assays. (E) The interactions between DCM1 and OsPABNs are verified by BiFC assay. The corresponding plasmid pairs were listed. CFP, cyan fluorescent protein. DIC, differential interference contrast micrographs.

To identify potential interacting partners of DCM1, we screened a rice anther cDNA library using the yeast two-hybrid system. The full-length DCM1 protein can autonomously activate the reporter gene in the absence of a prey protein and thus cannot be used as bait. Therefore, we split the DCM1 protein into two parts and tested their autoactivation activity separately. We found that the N terminus (1–1293) is responsible for autoactivation (Fig 8A). Therefore, the C terminus of DCM1 from the amino acid 1294 to the end was used as the bait.

From this screen, we identified the two nuclear poly (A) binding proteins (OsPABNs) in rice and named them OsPABN1 (LOC_Os02g52140) and OsPABN2 (LOC_Os06g11620) (32.7% of sequenced positive colonies), respectively (Fig 8B). OsPABN1 and OsPABN2 are homologs of human poly (A) binding protein nuclear 1 (PABPN1) and members of the polyadenylation factor family, which are required for mRNA polyadenylation. OsPABN1 and OsPABN2 share high sequence similarity (77% identity) and both contain an RNA recognition motif (RRM) in the central region. The clone with shortest sequence identified in this screen corresponds to a peptide ranging from the 85th amino acid to the C terminal end of OsPABN1, indicating that the coiled-coil domain is not necessary for its interaction with DCM1.

We conducted yeast two-hybrid assays to verify these interactions. Transformants with DCM1-C and OsPABN1, OsPABN2 grew on QDO/X/A media, confirming the interactions between them (Fig 8C). To clarify whether the tandem CCCH domain of DCM1 is required for its interaction with the OsPABNs, we split the DCM1-C into three parts, DCM1-D (1294–1563), DCM1-E (1557–1772), and DCM1-F (1773–2068) and tested their interactions with OsPABNs. The results showed that DCM1-D interacts with OsPABNs, while DCM1-E and DCM1-F, which contain the coiled-coil domain and the tandem CCCH domain, respectively, do not.

We also verified the interaction between DCM1 and the OsPABNs using the bimolecular fluorescence complementation (BiFC) assay (Fig 8E). Interactions between DCM1 and the two OsPABNs reconstituted the cyan fluorescent protein (CFP) in rice protoplasts and the CFP signal was observed in nuclear speckles. Moreover, OsPABN1 and OsPABN2 interact with themselves and between each other (Fig 8D and 8E). The BiFC results indicate that these interactions also take place primarily in the nuclear speckles.

### Expression level of genes involved in callose metabolism and exine development is altered in *dcm1*

**Fig 9:**
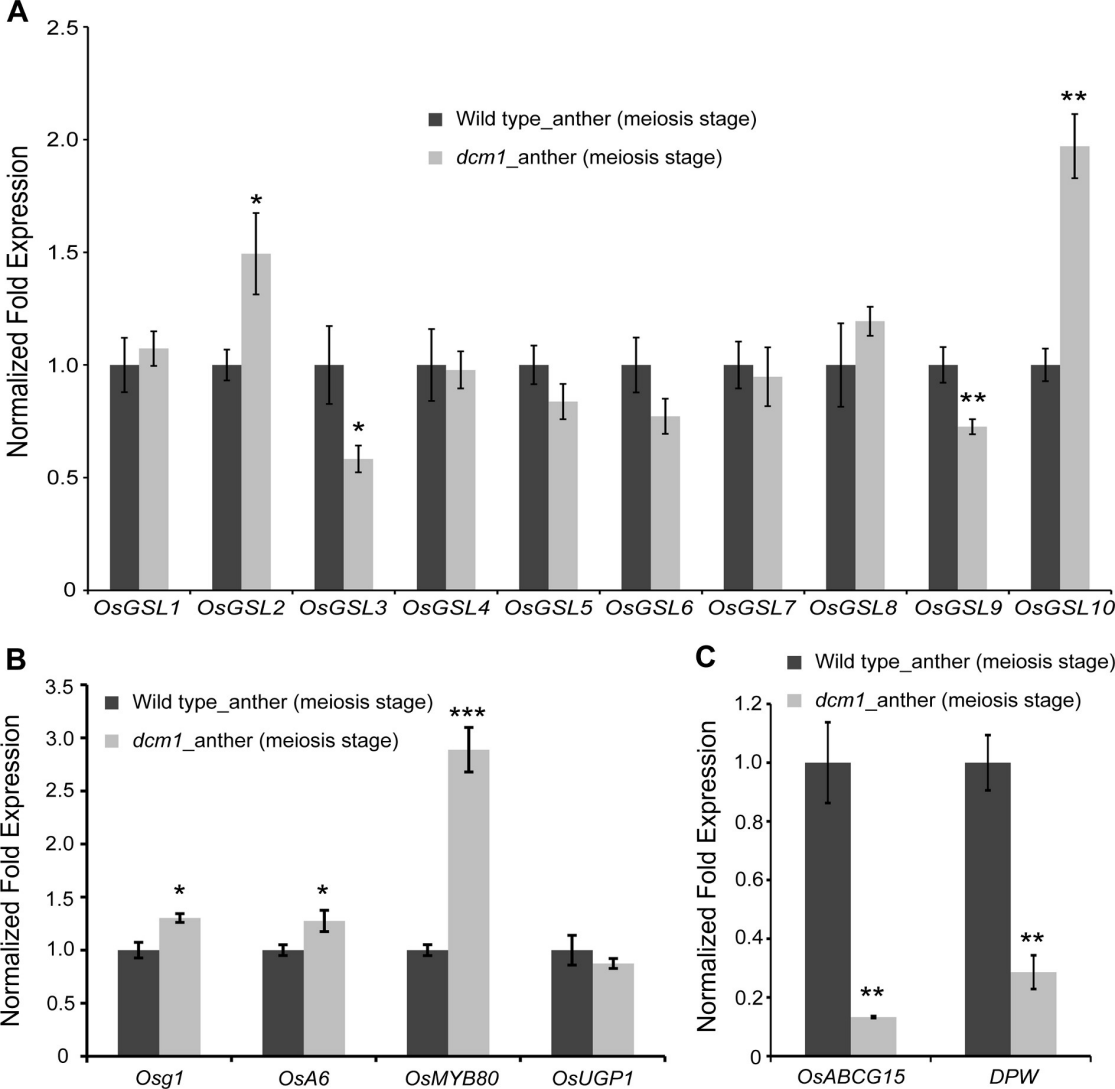
Expression analysis of genes involved in callose metabolism and exine formation in wild-type and *dcm1* anthers. (A) The expression comparison of *OsGSL1-OsGSL10* between wild-type and *dcm1* anthers during meiosis. (B) The expression comparison of *Osg1*, *OsA6*, *OsMYB80* and *OsUGP1* between wild-type and *dcm1* anthers during meiosis. (C) The expression comparison of *OsABCG15* and *DPW* between wild-type and *dcm1* anthers during meiosis. *Ubiquitin* is used as endogenous control. Error bars represent SD (n = 3). Asterisks indicate significant differences according to Student’s *t*-test (*P<0.05, **P<0.01 and ***P<0.001).

Based on the observation of prematurely dissolution of callose in the *dcm1* PMCs and DCM1 might be involved in mRNA metabolism, we examined the expression profiles of genes involved in callose metabolism in rice anthers during meiosis (Fig 9). There are ten predicted callose synthase genes (*OsGSL1-OsGSL10*) in the rice genome [29]. Among them, only *OsGSL5* and *OsGSL8* have been evaluated for their respective functions in male fertility and ovary expansion [14, 30]. Compared with wild type, the expression level of *OsGSL2* and *OsGSL10* increased by 1.49- and 1.97-fold, respectively, in *dcm1* (Fig 9A). However, the expression level of *OsGSL3* and *OsGSL9* in *dcm1* decreased to 58.2% and 72.6% of that in wild type, respectively. *OsGSL2* and *OsGSL3* are closely related to *AtGSL1* and *AtGSL5*, which synthesize the interstitial callose during meiosis in *Arabidopsis* [15]. No significant changes in expression level between wild type and *dcm1* were observed for the other *OsGSL* genes.

*Osg1* encodes a rice β-1, 3-glucanase that was reported to degrade callose during pollen development [31]. The expression level of this gene in *dcm1* increased to 1.30-fold of that in wild type (Fig 9B). The *A6* gene, which encodes an O-Glycosyl hydrolases family 17 protein, and its positive regulator *AtMYB80* were reported to affect callose dissolution in *Arabidopsis* [32, 33]. The expression patterns of their homologs in rice, *OsA6* and *OsMYB80*, were examined. In the mutant, the expression levels of *OsA6* increased to 1.28-fold and *OsMYB80* increased to 2.89-fold of wild type. These results suggested that callose degradation process might be altered in *dcm1*. No significant difference in the expression of *UGP1*, an UDP-glucose pyrophosphorylase gene that is essential for callose deposition [34], was observed between wild type and *dcm1*.

We also evaluated the expression level of genes related to exine formation (Fig 9C). *OsABCG15* encodes an ATP-binding cassette transporter protein that is essential for pollen exine development in rice [35]. The expression level of *OsABCG15* in *dcm1* reduced to 13.3% of that in wild type. A fatty acyl carrier protein reductase, DPW, plays a role in the formation of regular exine [36]. The expression level of *DPW* in the mutant reduced to 29.6% of that in wild type. These results suggested that exine development process is affected in *dcm1*. We also compared the expression level of genes related to meiosis. However, no significant differences in the expression of these genes, including *OsSPO11-1*, *OsCOM1*, *OsRAD17*, *OsDMC1*, *OsRAD51*, *OsMER3* and *OsREC8*, were observed between wild type and *dcm1* (S5 Fig).

## Discussion

In this study, we showed that dysfunction of DCM1, a tandem CCCH protein, disturbs the metabolism of callose both around the PMCs and at the cell plate. Without the callosic plate, meiotic cytokinesis in *dcm1* is not completed. The defective meiotic cytokinesis during telophase I lead to the random movement of the spindles at meiosis II and the varied arrangement of meiotic products. The aberrant meiotic products develop into pollen grains with varied sizes and DNA contents, which is reminiscent of the phenotypes of *atps1* and *jason* in *Arabidopsis* [27, 37]. In *Arabidopsis*, instead of the cell plate, an organelle band is formed during meiotic telophase I. The loss of AtPS1 or JASON protein leads to the disruption of organelle band, resulting in the disrupted spindle positioning during male meiosis II [38], just like that observed in *dcm1*.

### The role of callose in meiotic cytokinesis in monocotyledonous plants

The deposition of callose during mitotic and meiotic cytokinesis in plants has been meticulously analyzed by electron microscopy [39, 40]. It is possible that the membrane network serves as a trigger for the induction of callose accumulation, since it only appears directly at the cell plate and not in the secretory vesicles *en route* to the division plane [41]. Samuels *et al*. (1995) postulated that callose helps to mechanically stabilize the early delicate membrane networks of forming cell plates in tobacco, and to create a rapid spreading force that widens the tubules and converts them into plate-like structures. However, Thiele *et al*. (2009) revealed that the cell-plate membrane compartment forms and expands, seemingly as far as the parental wall, prior to the appearance of callose. Based on characterization of the *gsl8* mutant in *Arabidopsis*, they speculated that callose might be required for inserting the nascent crosswall at the division site during mitosis.

Callose is only a transient structure during somatic cytokinesis that is replaced by cellulose during the final step. However, this is not the case in successive-type meiotic cytokinesis, which is typically observed in monocots PMCs. Callosic plates are formed after both the first and the second caryokinesis events during meiosis. The callosic plate is preserved when the first cell division is completed and persists through the second cell division. Whether other polysaccharides, such as cellulose and pectin, are deposit on the cell plate during meiotic cytokinesis is still uncertain. It is possible that the callosic plate is the only physical barrier insulating the cytoplasm during meiotic cytokinesis.

### The possible mechanism by which DCM1 coordinates the metabolism of callose

The most prominent feature of DCM1 is the five tandem CCCH domain at its C-terminus, which is conserved between different kingdoms. The homolog of *DCM1* in *Arabidopsis* was named *SOP1* (*SUPPRESSOR OF PAS2 1*), the mutation of which suppressed the developmental defects of a splicing-defective allele of *PASTICCINO2* [42]. Red5, the homolog of DCM1 in fission yeast, is essential for efficient elimination of specific meiotic mRNAs during vegetative growth and the proper splicing of meiotic genes [43–45], indicating that DCM1 might be involved in mRNA processing or elimination. OsPABN1 and OsPABN2, the two nuclear poly A binding proteins in rice, were identified as interacting partners of DCM1. In fission yeast, Red5 was co-immunoprecipitated with Pab2, the orthologue of OsPABN1 and OsPABN2 [43, 46]. In addition, dZC3H3, the homolog of DCM1 in flies, also associates with PABP2 [47]. Recently, the direct interaction between Red5 and Pab2 was detected in a proteome-wide interactome study [48]. Therefore, the interactions between proteins containing five tandem CCCH domain and nuclear poly (A) binding proteins might be conserved in fungi, animals and plants.

Studies of DCM1 homologs in the other species mentioned above indicate that they are involved in nuclear exosome functions, and RNA degradation by the exosome is the main pathway for the removal of unwanted RNA in all kingdoms [49]. Moreover, studies in fission yeast have demonstrated that Pab2 and the nuclear exosome subunit, Rrp6, are the main factors involved in a polyadenylation-dependent pre-mRNA degradation pathway, and inefficient splicing is important to dictate susceptibility to this process [50]. The retained *PAS2* transcript in *sop1* harbors a nucleotide substitution before a GC splicing site that mimics a pre-mRNA with low splicing efficiency [42], indicating that the tandem CCCH zinc finger protein and the poly (A) binding protein could be involved in conserved biological processes across species.

During meiosis, callose metabolism must be precisely controlled to ensure the completion of meiotic cytokinesis by its deposition and release of microspores by its dissolution. In *dcm1*, callose around PMCs was deposited normally during leptotene and disappeared during pachytene (Fig 4B). Similarly, prematurely dissolution of callosic plates occurred during telophase I and telophase II in the mutant. It seems that the dynamic changes in callose (deposition and dissolution) in *dcm1* reflect the antagonism between callose synthesis and degradation. We speculate that the defective callose metabolism in *dcm1* is caused by unwanted RNAs that are eliminated in the presence of DCM1. In *Arabidopsis*, the expression of some callose-related genes is affected by the mutation of *CALLOSE DEFECTIVE MICROSPORE1* (*CDM1*), a gene encoding a tandem CCCH-type zinc finger protein [51]. *CalS5*, the *Arabidopsis* gene responsible for callose deposition surrounding the PMCs, tetrads and microspores, is regulated in a post-transcription manner [52]. We checked the expression level, splicing and polyadenylation site of *OsGSL5* gene and no significant differences were found between *dcm1* and wild type. Besides *Osg1*, whose function was demonstrated by RNA interference assay [31], few callase genes (encoding β-1, 3-glucanase) have been functionally characterized in rice meiosis. Thus, further studies to identify the mRNA targets of DCM1 will help to uncover the mechanisms by which DCM1 regulates callose metabolism during rice meiosis.

## Materials and methods

### Plant materials and grow conditions

The *dcm1* mutant was isolated from a collection of mutants induced by ^60^Co-Ƴ-ray irradiation on *indica* rice (*Oryza sativa*) variety, Guangluai 4. The variety Guangluai 4 was used as the wild type in all experiments. All plants were grown in the paddy field.

### Map-based cloning of the *DCM1* gene

When a line segregated 1:3 for sterile and fertile plants, the fertile plants was selected to cross with the *japonica* rice variety Wuyunjing 8. Using the sterile plants that segregated in the F2, F3 and F4 population (299 plants in total), the mutated gene was mapped to a 138.5kb interval between markers C6-25.86 and C6-26.003. Based on the MSU Rice Genome Annotation Project Database and Resource (http://rice.plantbiology.msu.edu/), there are 23 predicted genes in this region and 1bp deletion in the gene *LOC4341610* was detected between the mutant and wild type by sequencing. Indel (insertion-deletion) markers used for mapping were designed based on the sequence differences between *indica* variety 9311 and *japonica* variety Nipponbare according to the data published (http://www.ncbi.nlm.nih.gov). Primers used were listed in the supporting information (S2 Table).

### Knock out *DCM1* by CRISPR/Cas9 gene editing approach

The CRISPR/Cas9 gene editing approach in rice was performed according to a protocol described previously [53]. Target sequences (TTGGATTTGACTTTGCTCTT) were selected according to the following criterions: (1) Sequence located in the exon. (2) Followed by the PAM (NGG). (3) Be unique among the genome to avoid multiple knock out sites. Designed primer were incubated at 100°C for 5 min and subcloned into vector pC1300-Cas9-1gRNA with a hygromycin resistance marker. The resulting constructs were transformed into *Agrobacterium tumefaciens* EHA105 and then into calli of Yandao 8, a *japonica* variety. T_0_ plants were genotyped to verify whether the editing site was modified. Target sequences used in this study were listed in the supporting information (S2 Table).

### Full-length cDNA cloning of *DCM1*

Total RNA extraction was conducted using the TRIzol reagent (Invitrogen), as described by the supplier, from rice young panicle. Reverse transcription was performed using SuperScript III First-Strand Synthesis System for RT-PCR kit (Invitrogen). The cDNA was amplified using the 5’and 3’-Full RACE kits (TaKaRa). PCR using primers CDS-F and CDS-R was performed to amplify the open reading frame. The sequences were sequenced and then spliced together to obtain the full-length cDNA. Primers mentioned above were listed in the supporting information (S2 Table).

### Quantitative RT-PCR assay, *promoter*::*GUS* analysis of *DCM1* expression pattern and RNA *in situ* hybridization

Real-time PCR analysis was performed using the Bio-Rad CFX96 real-time PCR instrument and Hieff qPCR SYBR Green Master Mix (No Rox Plus) (YEASEN). All PCR experiments were conducted using 40 cycles of 95°C for 10 s, 60°C for 30 s. All reactions were performed in triplicate, with *Ubiqutin* as the normalized reference gene for all comparisons. The primers for qRT-PCR were listed in S2 Table.

A 2.4Kb upstream region of the *DCM1* gene was amplified and cloned into pCAMBIA1301. The construct was introduced into the *japonica* rice variety Nipponbare. Spikelets at different developmental stages from transgenic lines were incubated with X-Gluc (5-bromo-4-chloro-3-indolyl-β-D-glucuronide) solution. The spikelets were cleared in 75% (v/v) ethanol and photographed with a stereoscope.

For *in situ* hybridization, sense and antisense probes were synthesized with T7 RNA polymerase using the digoxigenin RNA labeling kit (Roche). Tissue fixation and hybridization were performed as previously described [54]. Primers used were listed in S2 Table.

### Cytology

Rice anthers at various developmental stages were fixed in 4% paraformaldehyde overnight. The samples were washed in PBS, dehydrated in a graded ethanol series, and embedded in Technovit 7100 resin (Heraeus Kulzer). Microtome sections (4μm thick) were stained with 0.25% toluidine blue to stain the cells and 0.1% aniline blue to stain the callose, respectively. The slides were photographed with an Olympus BX51 microscope and a digital camera.

DAPI staining of mature pollen was performed according to Liu [55]. Young panicles were fixed in Carnoy Solution for at least 24 h at room temperature. Anthers at the proper developmental stage were then squashed in acetocarmine. Meiotic chromosome preparation and immunofluorescence analysis were performed as previously described [56]. Aniline blue (0.1% in PBS) was applied to slides with DAPI to co-stain the callose and DNA. Images were captured under a Zeiss A2 fluorescence microscope with a micro CCD camera. Immunogold assay was conducted using antibody to β-1, 3-glucan at dilution of 1:100 (Biosupplies). The secondary antibody is goat anti-mouse IgG-15 nm gold (Abcam) at dilution of 1:20.

### Two-hybrid cDNA library screening and yeast two-hybrid (Y2H) assay

Total RNA was extracted from wild-type anthers during meiosis. Highly purified and intact mRNA was isolated using mRNA Purification Kit (Life technology, NO. 61006). cDNA library construction was performed using Make Your Own Mate & Plate Library System (Clontech NO.630490) according to the manufacturer’s instructions. The Y2H assay was conducted with Yeast Transformation System 2 (Clontech NO.630439). The yeast strain Y2HGOLD was used for library screening and Y2H assays. All primers used were listed in S2 Table.

### BIFC assay

The BiFC assay was performed as previously described [57]. The full-length coding sequences of *DCM1*, *OsPABN1* and *OsPABN2* were subcloned into vector pSCYNE(R) and pSCYCE(R), respectively. The plasmid pairs were co-transfected into protoplasts of young rice seedling by 40% PEG (polyethylene glycol). Transfected protoplasts were incubated in the dark at 28°C overnight, and observed using a laser scanning confocal microscopy. Primers used were listed in S2 Table.

## Supporting information

S1 Fig The microspores of *dcm1* vary in size. (A), Comparison of microspores between wild type and *dcm1*. Bars = 20μm. (B), Distribution of microspores size in wild type and *dcm1*.
(TIF)

S2 Fig The formation of callosic plate in wild type PMCs. (A), Anther before callosic plate formation. (B), Anther during callosic plate formation. (C), Anther after callosic plate formation. Callosic plates are indicated by arrows. Bars = 10μm.
(TIF)

S3 Fig Map-based cloning of *DCM1*. Horizontal lines, *DCM1*-located region; vertical lines, markers and numbers of recombinants. The genes located in the mapped region are depicted in boxes. *DCM1* gene (*LOC4341610*) is showed in red box.
(TIF)

S4 Fig Knocking out the *DCM1* gene in wild-type plants disrupted meiotic cytokinesis. (A), The mutated site is located before the CCCH domain. (B), Details of the mutated site. Inserted nucleotide is shown in red. (C), Meiotic cytokinesis is defective in *dcm1-cas9* transgene line. Bars = 10um.
(TIF)

S5 Fig Expression analysis of genes involved in meiosis in wild-type and *dcm1* anther. *Ubiquitin* is used as endogenous control. Error bars represent SD (n = 3). n.s., no significant differences, *P*>0.05 in two-tailed Student’s *t*-tests.
(TIF)

S1 Table Seed setting rate of wild type and *dcm1* when pollinated with wild-type pollen grains. (DOCX)

S2 Table Primers used in this study. (DOCX)

## References

[1] Balasubramanian MK, Bi E, Glotzer M. Comparative analysis of cytokinesis in budding yeast, fission yeast and animal cells. Curr Biol. 2004;14(18):R806–18. 10.1016/j.cub.2004.09.022 . 15380095 10.1016/j.cub.2004.09.022

[2] Jurgens G. Cytokinesis in higher plants. Annu Rev Plant Biol. 2005;56:281–99. 10.1146/annurev.arplant.55.031903.141636 . 15862097 10.1146/annurev.arplant.55.031903.141636

[3] Steiner A, Muller L, Rybak K, Vodermaier V, Facher E, Thellmann M, et al The Membrane-Associated Sec1/Munc18 KEULE is Required for Phragmoplast Microtubule Reorganization During Cytokinesis in Arabidopsis. Mol Plant. 2016;9(4):528–40. 10.1016/j.molp.2015.12.005 . 26700031 10.1016/j.molp.2015.12.005

[4] Zickler D, Kleckner N. Meiotic chromosomes: integrating structure and function. Annu Rev Genet. 1999;33:603–754. 10.1146/annurev.genet.33.1.603 . 10690419 10.1146/annurev.genet.33.1.603

[5] De Storme N, Geelen D. Cytokinesis in plant male meiosis. Plant Signal Behav. 2013;8(3):e23394 10.4161/psb.23394 ; PubMed Central PMCID: PMCPMC3676507. 23333967 10.4161/psb.23394 PMC3676507

[6] Chen XY, Kim JY. Callose synthesis in higher plants. Plant Signal Behav. 2009;4(6):489–92. 10.4161/psb.4.6.8359 ; PubMed Central PMCID: PMCPMC2688293. 19816126 10.4161/psb.4.6.8359 PMC2688293

[7] Cheng X, Zhu L, He G. Towards understanding of molecular interactions between rice and the brown planthopper. Mol Plant. 2013;6(3):621–34. 10.1093/mp/sst030 23396040 10.1093/mp/sst030

[8] Samuels AL, Giddings TH Jr., Staehelin LA. Cytokinesis in tobacco BY-2 and root tip cells: a new model of cell plate formation in higher plants. J Cell Biol. 1995;130(6):1345–57. ; PubMed Central PMCID: PMCPMC2120572. 7559757 10.1083/jcb.130.6.1345 PMC2120572

[9] Chen XY, Liu L, Lee E, Han X, Rim Y, Chu H, et al The Arabidopsis callose synthase gene GSL8 is required for cytokinesis and cell patterning. Plant Physiol. 2009;150(1):105–13. 10.1104/pp.108.133918 ; PubMed Central PMCID: PMCPMC2675722. 19286936 10.1104/pp.108.133918 PMC2675722

[10] Thiele K, Wanner G, Kindzierski V, Jurgens G, Mayer U, Pachl F, et al The timely deposition of callose is essential for cytokinesis in *Arabidopsis*. Plant J. 2009;58(1):13–26. 10.1111/j.1365-313X.2008.03760.x . 19067977 10.1111/j.1365-313X.2008.03760.x

[11] De Storme N, De Schrijver J, Van Criekinge W, Wewer V, Dormann P, Geelen D. GLUCAN SYNTHASE-LIKE8 and STEROL METHYLTRANSFERASE2 are required for ploidy consistency of the sexual reproduction system in *Arabidopsis*. Plant Cell. 2013;25(2):387–403. 10.1105/tpc.112.106278 ; PubMed Central PMCID: PMCPMC3608767. 23404886 10.1105/tpc.112.106278 PMC3608767

[12] Dong X, Hong Z, Sivaramakrishnan M, Mahfouz M, Verma DP. Callose synthase (CalS5) is required for exine formation during microgametogenesis and for pollen viability in *Arabidopsis*. Plant J. 2005;42(3):315–28. 10.1111/j.1365-313X.2005.02379.x . 15842618 10.1111/j.1365-313X.2005.02379.x

[13] Nishikawa S, Zinkl GM, Swanson RJ, Maruyama D, Preuss D. Callose (beta-1,3 glucan) is essential for Arabidopsis pollen wall patterning, but not tube growth. Bmc Plant Biol. 2005;5:22 10.1186/1471-2229-5-22 ; PubMed Central PMCID: PMCPMC1274334. 16212660 10.1186/1471-2229-5-22 PMC1274334

[14] Shi X, Sun X, Zhang Z, Feng D, Zhang Q, Han L, et al GLUCAN SYNTHASE-LIKE 5 (GSL5) plays an essential role in male fertility by regulating callose metabolism during microsporogenesis in rice. Plant Cell Physiol. 2015;56(3):497–509. 10.1093/pcp/pcu193 . 25520407 10.1093/pcp/pcu193

[15] Enns LC, Kanaoka MM, Torii KU, Comai L, Okada K, Cleland RE. Two callose synthases, GSL1 and GSL5, play an essential and redundant role in plant and pollen development and in fertility. Plant Mol Biol. 2005;58(3):333–49. 10.1007/s11103-005-4526-7 . 16021399 10.1007/s11103-005-4526-7

[16] Zeng Q, Chen JG, Ellis BE. AtMPK4 is required for male-specific meiotic cytokinesis in *Arabidopsis*. Plant J. 2011;67(5):895–906. 10.1111/j.1365-313X.2011.04642.x . 21575092 10.1111/j.1365-313X.2011.04642.x

[17] Spielman M, Preuss D, Li FL, Browne WE, Scott RJ, Dickinson HG. TETRASPORE is required for male meiotic cytokinesis in Arabidopsis thaliana. Development. 1997;124(13):2645–57. . 9217006 10.1242/dev.124.13.2645

[18] Yang CY, Spielman M, Coles JP, Li Y, Ghelani S, Bourdon V, et al *TETRASPORE* encodes a kinesin required for male meiotic cytokinesis in *Arabidopsis*. Plant J. 2003;34(2):229–40. . 12694597 10.1046/j.1365-313x.2003.01713.x

[19] Wang D, Guo Y, Wu C, Yang G, Li Y, Zheng C. Genome-wide analysis of CCCH zinc finger family in *Arabidopsis* and rice. Bmc Genomics. 2008;9:44 10.1186/1471-2164-9-44 ; PubMed Central PMCID: PMCPMC2267713. 18221561 10.1186/1471-2164-9-44 PMC2267713

[20] Peng X, Zhao Y, Cao J, Zhang W, Jiang H, Li X, et al CCCH-type zinc finger family in maize: genome-wide identification, classification and expression profiling under abscisic acid and drought treatments. PLoS One. 2012;7(7):e40120 10.1371/journal.pone.0040120 ; PubMed Central PMCID: PMCPMC3391233. 22792223 10.1371/journal.pone.0040120 PMC3391233

[21] Zhang C, Zhang H, Zhao Y, Jiang H, Zhu S, Cheng B, et al Genome-wide analysis of the CCCH zinc finger gene family in *Medicago truncatula*. Plant Cell Rep. 2013;32(10):1543–55. 10.1007/s00299-013-1466-6 . 23749175 10.1007/s00299-013-1466-6

[22] Pradhan S, Kant C, Verma S, Bhatia S. Genome-wide analysis of the CCCH zinc finger family identifies tissue specific and stress responsive candidates in chickpea (*Cicer arietinum L*.). PLoS One. 2017;12(7):e0180469 10.1371/journal.pone.0180469 ; PubMed Central PMCID: PMCPMC5507508. 28704400 10.1371/journal.pone.0180469 PMC5507508

[23] Bogamuwa SP, Jang JC. Tandem CCCH zinc finger proteins in plant growth, development and stress response. Plant Cell Physiol. 2014;55(8):1367–75. 10.1093/pcp/pcu074 . 24850834 10.1093/pcp/pcu074

[24] Zhang D, Xu Z, Cao S, Chen K, Li S, Liu X, et al An uncanonical CCCH-tandem zinc-finger protein represses secondary wall synthesis and controls mechanical strength in rice. Mol Plant. 2018;11(1):163–74. 10.1016/j.molp.2017.11.004 . 29175437 10.1016/j.molp.2017.11.004

[25] Jan A, Maruyama K, Todaka D, Kidokoro S, Abo M, Yoshimura E, et al OsTZF1, a CCCH-tandem zinc finger protein, confers delayed senescence and stress tolerance in rice by regulating stress-related genes. Plant Physiol. 2013;161(3):1202–16. 10.1104/pp.112.205385 ; PubMed Central PMCID: PMCPMC3585590. 23296688 10.1104/pp.112.205385 PMC3585590

[26] Qu J, Kang SG, Wang W, Musier-Forsyth K, Jang JC. The Arabidopsis thaliana tandem zinc finger 1 (AtTZF1) protein in RNA binding and decay. Plant J. 2014;78(3):452–67. 10.1111/tpj.12485 ; PubMed Central PMCID: PMCPMC4026020. 24635033 10.1111/tpj.12485 PMC4026020

[27] De Storme N, Geelen D. The *Arabidopsis* mutant *jason* produces unreduced first division restitution male gametes through a parallel/fused spindle mechanism in meiosis II. Plant Physiol. 2011;155(3):1403–15. 10.1104/pp.110.170415 ; PubMed Central PMCID: PMCPMC3046594. 21257792 10.1104/pp.110.170415 PMC3046594

[28] Zhang Z, Lu Y, Liu X, Feng J. Dynamic changes of callose in microsporogenesis and microgametogenesis of rice (Oryza sativa L.). Acta Agronomica Sinica. 2007.

[29] Yamaguchi T, Hayashi T, Nakayama K, Koike S. Expression analysis of genes for callose synthases and Rho-type small GTP-binding proteins that are related to callose synthesis in rice anther. Biosci Biotechnol Biochem. 2006;70(3):639–45. 10.1271/bbb.70.639 . 16556979 10.1271/bbb.70.639

[30] Song L, Wang R, Zhang L, Wang Y, Yao S. CRR1 encoding callose synthase functions in ovary expansion by affecting vascular cell patterning in rice. Plant J. 2016;88(4):620–32. 10.1111/tpj.13287 . 27464824 10.1111/tpj.13287

[31] Wan L, Zha W, Cheng X, Liu C, Lv L, Liu C, et al A rice beta-1,3-glucanase gene *Osg1* is required for callose degradation in pollen development. Planta. 2011;233(2):309–23. 10.1007/s00425-010-1301-z . 21046148 10.1007/s00425-010-1301-z

[32] Hird DL, Worrall D, Hodge R, Smartt S, Paul W, Scott R. The anther-specific protein encoded by the Brassica napus and Arabidopsis thaliana A6 gene displays similarity to beta-1,3-glucanases. Plant J. 1993;4(6):1023–33. . 8281185 10.1046/j.1365-313x.1993.04061023.x

[33] Zhang ZB, Zhu J, Gao JF, Wang C, Li H, Li H, et al Transcription factor AtMYB103 is required for anther development by regulating tapetum development, callose dissolution and exine formation in Arabidopsis. Plant J. 2007;52(3):528–38. 10.1111/j.1365-313X.2007.03254.x . 17727613 10.1111/j.1365-313X.2007.03254.x

[34] Chen R, Zhao X, Shao Z, Wei Z, Wang Y, Zhu L, et al Rice UDP-glucose pyrophosphorylase1 is essential for pollen callose deposition and its cosuppression results in a new type of thermosensitive genic male sterility. Plant Cell. 2007;19(3):847–61. 10.1105/tpc.106.044123 ; PubMed Central PMCID: PMCPMC1867369. 17400897 10.1105/tpc.106.044123 PMC1867369

[35] Qin P, Tu B, Wang Y, Deng L, Quilichini TD, Li T, et al *ABCG15* encodes an ABC transporter protein, and is essential for post-meiotic anther and pollen exine development in rice. Plant Cell Physiol. 2013;54(1):138–54. 10.1093/pcp/pcs162 . 23220695 10.1093/pcp/pcs162

[36] Shi J, Tan H, Yu XH, Liu Y, Liang W, Ranathunge K, et al *Defective pollen wall* is required for anther and microspore development in rice and encodes a fatty acyl carrier protein reductase. Plant Cell. 2011;23(6):2225–46. 10.1105/tpc.111.087528 ; PubMed Central PMCID: PMCPMC3160036. 21705642 10.1105/tpc.111.087528 PMC3160036

[37] d'Erfurth I, Jolivet S, Froger N, Catrice O, Novatchkova M, Simon M, et al Mutations in *AtPS1* (*Arabidopsis thaliana parallel spindle 1*) lead to the production of diploid pollen grains. PLoS Genet. 2008;4(11):e1000274 10.1371/journal.pgen.1000274 ; PubMed Central PMCID: PMCPMC2581889 pollen; 08290672.8). 19043546 10.1371/journal.pgen.1000274 PMC2581889

[38] Brownfield L, Yi J, Jiang H, Minina EA, Twell D, Kohler C. Organelles maintain spindle position in plant meiosis. Nat Commun. 2015;6:6492 10.1038/ncomms7492 . 25757555 10.1038/ncomms7492

[39] Segui-Simarro JM, Austin JR 2nd, White EA, Staehelin LA. Electron tomographic analysis of somatic cell plate formation in meristematic cells of Arabidopsis preserved by high-pressure freezing. Plant Cell. 2004;16(4):836–56. 10.1105/tpc.017749 ; PubMed Central PMCID: PMCPMC412860. 15020749 10.1105/tpc.017749 PMC412860

[40] Otegui MS, Staehelin LA. Electron tomographic analysis of post-meiotic cytokinesis during pollen development in *Arabidopsis thaliana*. Planta. 2004;218(4):501–15. 10.1007/s00425-003-1125-1 . 14610676 10.1007/s00425-003-1125-1

[41] Drakakaki G. Polysaccharide deposition during cytokinesis: challenges and future perspectives. Plant Sci. 2015;236:177–84. 10.1016/j.plantsci.2015.03.018 . 26025531 10.1016/j.plantsci.2015.03.018

[42] Hematy K, Bellec Y, Podicheti R, Bouteiller N, Anne P, Morineau C, et al The zinc-finger protein SOP1 is required for a subset of the nuclear exosome functions in *Arabidopsis*. PLoS Genet. 2016;12(2):e1005817 10.1371/journal.pgen.1005817 ; PubMed Central PMCID: PMCPMC4735120. 26828932 10.1371/journal.pgen.1005817 PMC4735120

[43] Sugiyama T, Wanatabe N, Kitahata E, Tani T, Sugioka-Sugiyama R. Red5 and three nuclear pore components are essential for efficient suppression of specific mRNAs during vegetative growth of fission yeast. Nucleic Acids Res. 2013;41(13):6674–86. 10.1093/nar/gkt363 ; PubMed Central PMCID: PMCPMC3711435. 23658229 10.1093/nar/gkt363 PMC3711435

[44] Zhou Y, Zhu J, Schermann G, Ohle C, Bendrin K, Sugioka-Sugiyama R, et al The fission yeast MTREC complex targets CUTs and unspliced pre-mRNAs to the nuclear exosome. Nat Commun. 2015;6:7050 10.1038/ncomms8050 ; PubMed Central PMCID: PMCPMC4455066. 25989903 10.1038/ncomms8050 PMC4455066

[45] Marayati BF, Hoskins V, Boger RW, Tucker JF, Fishman ES, Bray AS, et al The fission yeast MTREC and EJC orthologs ensure the maturation of meiotic transcripts during meiosis. RNA. 2016;22(9):1349–59. 10.1261/rna.055608.115 ; PubMed Central PMCID: PMCPMC4986891. 27365210 10.1261/rna.055608.115 PMC4986891

[46] Egan ED, Braun CR, Gygi SP, Moazed D. Post-transcriptional regulation of meiotic genes by a nuclear RNA silencing complex. RNA. 2014;20(6):867–81. 10.1261/rna.044479.114 ; PubMed Central PMCID: PMCPMC4024641. 24713849 10.1261/rna.044479.114 PMC4024641

[47] Hurt JA, Obar RA, Zhai B, Farny NG, Gygi SP, Silver PA. A conserved CCCH-type zinc finger protein regulates mRNA nuclear adenylation and export. J Cell Biol. 2009;185(2):265–77. 10.1083/jcb.200811072 ; PubMed Central PMCID: PMCPMC2700372. 19364924 10.1083/jcb.200811072 PMC2700372

[48] Vo TV, Das J, Meyer MJ, Cordero NA, Akturk N, Wei X, et al A proteome-wide fission yeast interactome reveals network evolution principles from yeasts to human. Cell. 2016;164(1–2):310–23. 10.1016/j.cell.2015.11.037 ; PubMed Central PMCID: PMCPMC4715267. 26771498 10.1016/j.cell.2015.11.037 PMC4715267

[49] Houseley J, LaCava J, Tollervey D. RNA-quality control by the exosome. Nat Rev Mol Cell Biol. 2006;7(7):529–39. 10.1038/nrm1964 . 16829983 10.1038/nrm1964

[50] Lemieux C, Marguerat S, Lafontaine J, Barbezier N, Bahler J, Bachand F. A pre-mRNA degradation pathway that selectively targets intron-containing genes requires the nuclear poly(A)-binding protein. Mol Cell. 2011;44(1):108–19. 10.1016/j.molcel.2011.06.035 . 21981922 10.1016/j.molcel.2011.06.035

[51] Lu P, Chai M, Yang J, Ning G, Wang G, Ma H. The *Arabidopsis CALLOSE DEFECTIVE MICROSPORE 1* gene is required for male fertility through regulating callose metabolism during microsporogenesis. Plant Physiol. 2014;164(4):1893–904. 10.1104/pp.113.233387 ; PubMed Central PMCID: PMCPMC3982751. 24567187 10.1104/pp.113.233387 PMC3982751

[52] Huang XY, Niu J, Sun MX, Zhu J, Gao JF, Yang J, et al CYCLIN-DEPENDENT KINASE G1 is associated with the spliceosome to regulate CALLOSE SYNTHASE5 splicing and pollen wall formation in *Arabidopsis*. Plant Cell. 2013;25(2):637–48. 10.1105/tpc.112.107896 ; PubMed Central PMCID: PMCPMC3608783. 23404887 10.1105/tpc.112.107896 PMC3608783

[53] Wang C, Shen L, Fu Y, Yan C, Wang K. A Simple CRISPR/Cas9 System for Multiplex Genome Editing in Rice. J Genet Genomics. 2015;42(12):703–6. 10.1016/j.jgg.2015.09.011 . 26743988 10.1016/j.jgg.2015.09.011

[54] Li GS, Meng Z, Kong HZ, Chen ZD, Theissen G, Lu AM. Characterization of candidate class A, B and E floral homeotic genes from the perianthless basal angiosperm Chloranthus spicatus (Chloranthaceae). Dev Genes Evol. 2005;215(9):437–49. 10.1007/s00427-005-0002-2 . 16028057 10.1007/s00427-005-0002-2

[55] Liu L, Zheng C, Kuang B, Wei L, Yan L, Wang T. Receptor-Like Kinase RUPO Interacts with Potassium Transporters to Regulate Pollen Tube Growth and Integrity in Rice. PLoS Genet. 2016;12(7):e1006085 10.1371/journal.pgen.1006085 ; PubMed Central PMCID: PMCPMC4957769. 27447945 10.1371/journal.pgen.1006085 PMC4957769

[56] Shen Y, Tang D, Wang K, Wang M, Huang J, Luo W, et al ZIP4 in homologous chromosome synapsis and crossover formation in rice meiosis. J Cell Sci. 2012;125(Pt 11):2581–91. 10.1242/jcs.090993 . 22393242 10.1242/jcs.090993

[57] Waadt R, Schmidt LK, Lohse M, Hashimoto K, Bock R, Kudla J. Multicolor bimolecular fluorescence complementation reveals simultaneous formation of alternative CBL/CIPK complexes in planta. Plant J. 2008;56(3):505–16. 10.1111/j.1365-313X.2008.03612.x . 18643980 10.1111/j.1365-313X.2008.03612.x

