# Supplementary figures and images for: The zinc finger protein DCM1 is required for male meiotic cytokinesis by preserving callose in rice
Source: PLoS Genet. 2018 Nov 12;14(11):e1007769. doi: 10.1371/journal.pgen.1007769 (PMC6258382; doi:10.1371/journal.pgen.1007769)

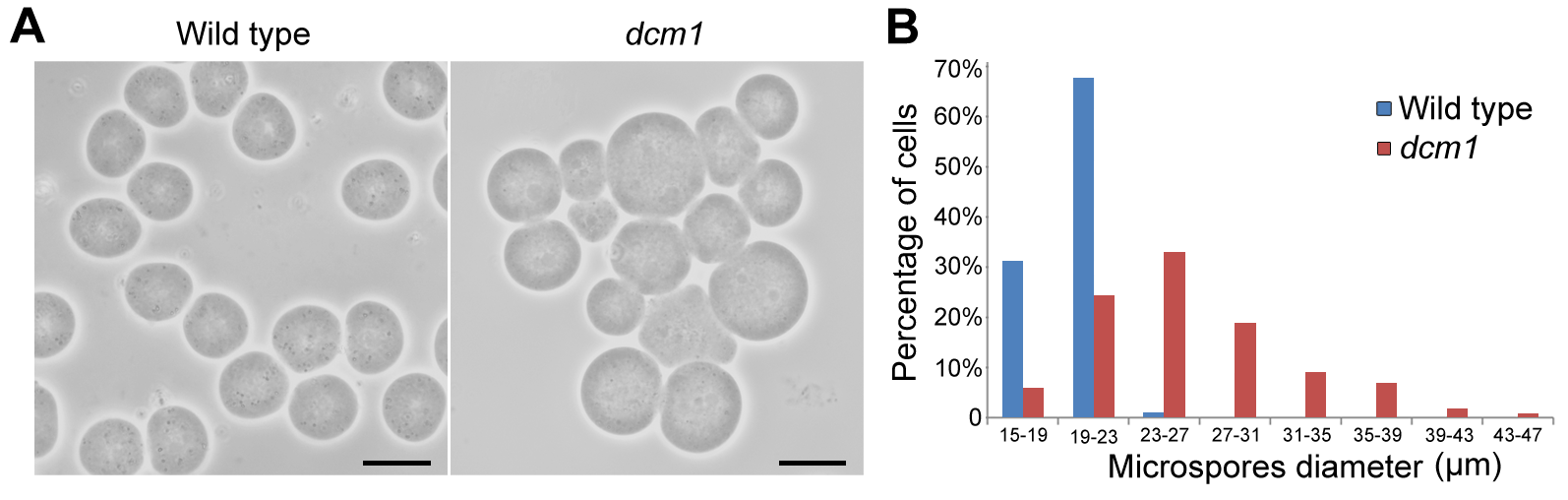

Supplement: S1 Fig — (A), Comparison of microspores between wild type and dcm1. Bars = 20μm. (B), Distribution of microspores size in wild type and dcm1. (TIF) [file pgen.1007769.s001.tif]

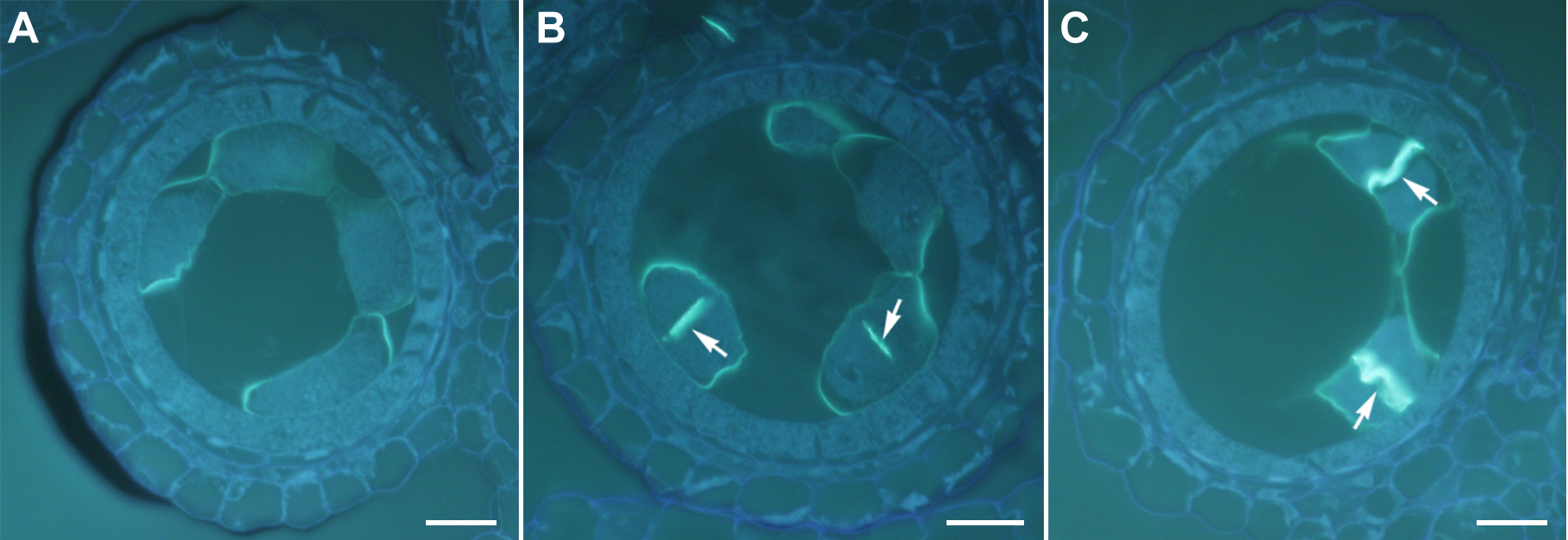

Supplement: S2 Fig — (A), Anther before callosic plate formation. (B), Anther during callosic plate formation. (C), Anther after callosic plate formation. Callosic plates are indicated by arrows. Bars = 10μm. (TIF) [file pgen.1007769.s002.tif]

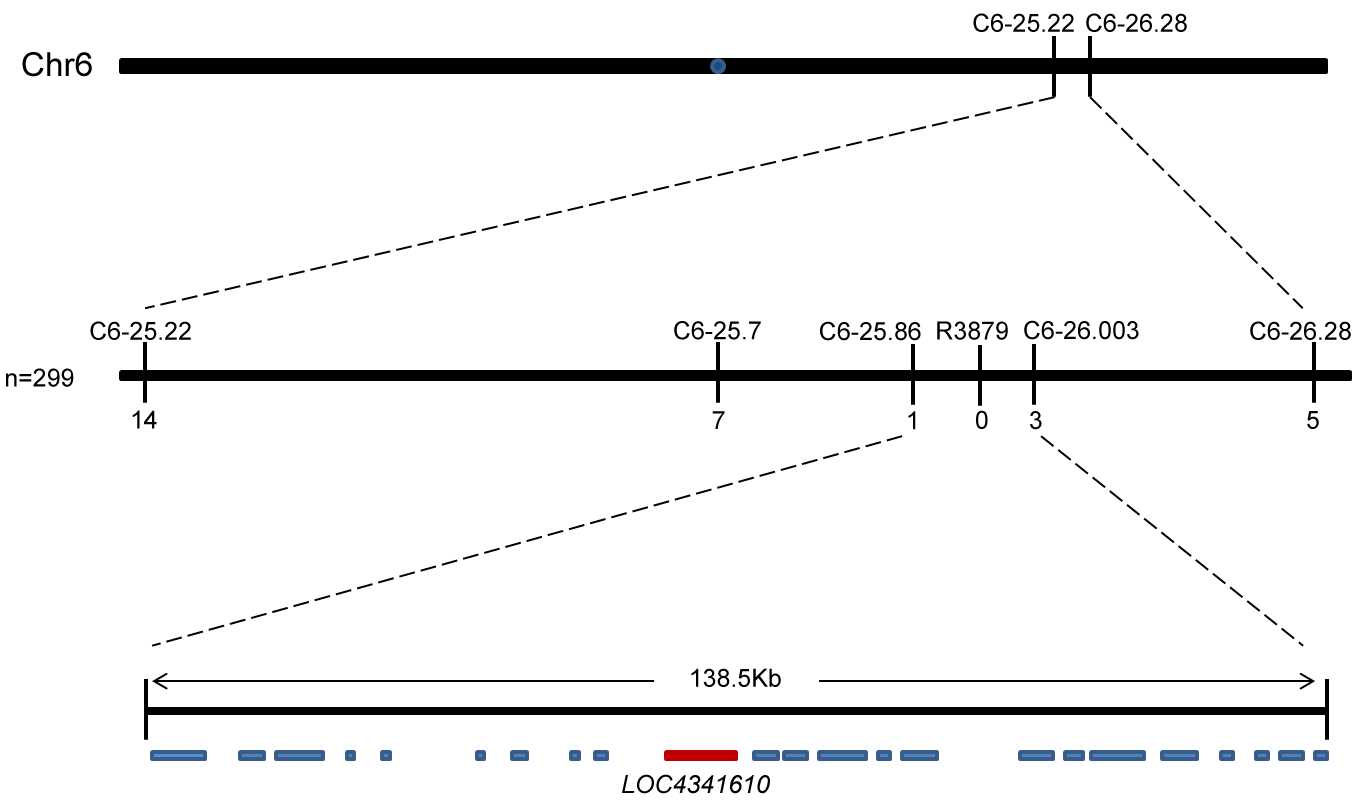

Supplement: S3 Fig — Horizontal lines, DCM1-located region; vertical lines, markers and numbers of recombinants. The genes located in the mapped region are depicted in boxes. DCM1 gene (LOC4341610) is showed in red box. (TIF) [file pgen.1007769.s003.tif]

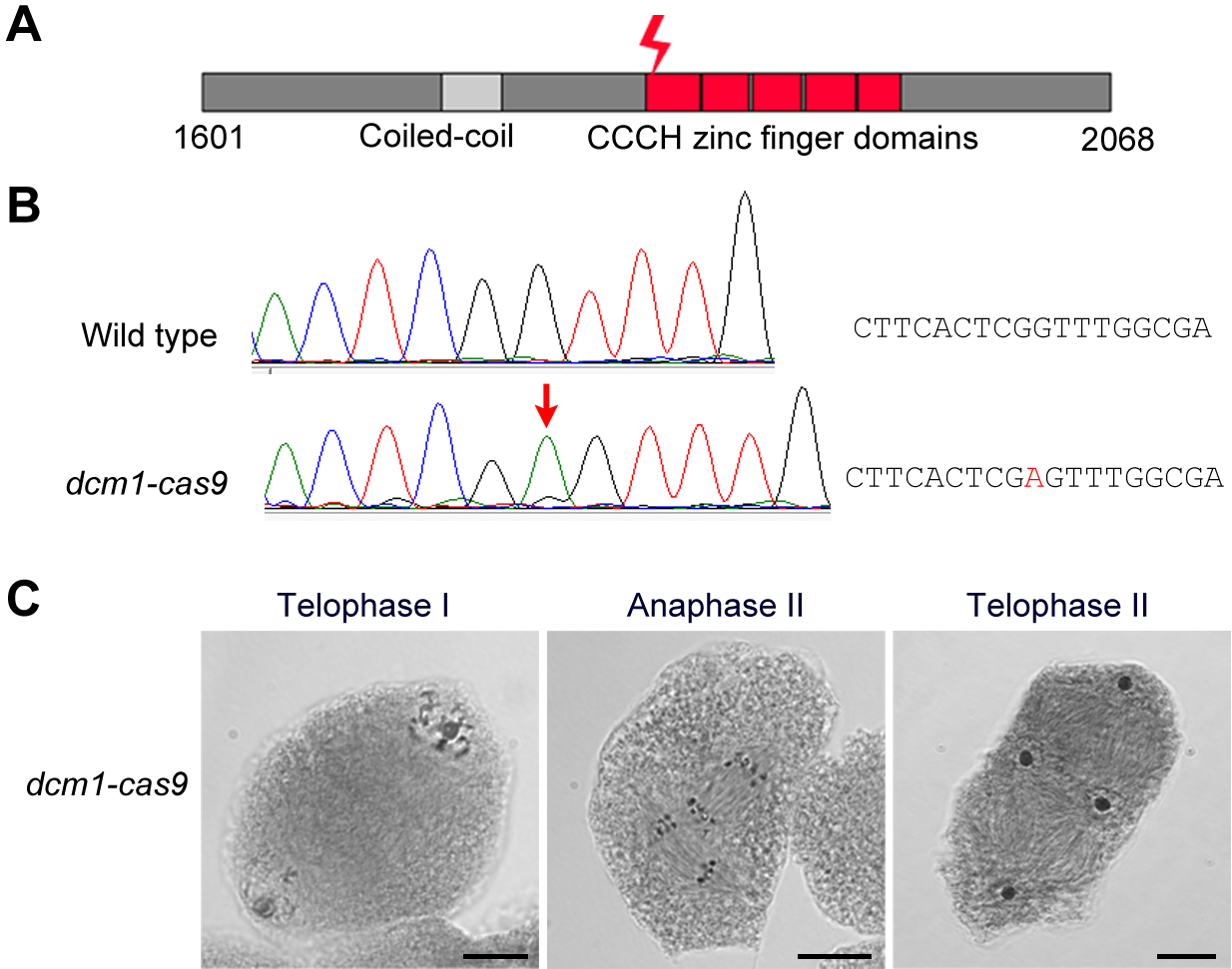

Supplement: S4 Fig — (A), The mutated site is located before the CCCH domain. (B), Details of the mutated site. Inserted nucleotide is shown in red. (C), Meiotic cytokinesis is defective in dcm1-cas9 transgene line. Bars = 10um. (TIF) [file pgen.1007769.s004.tif]

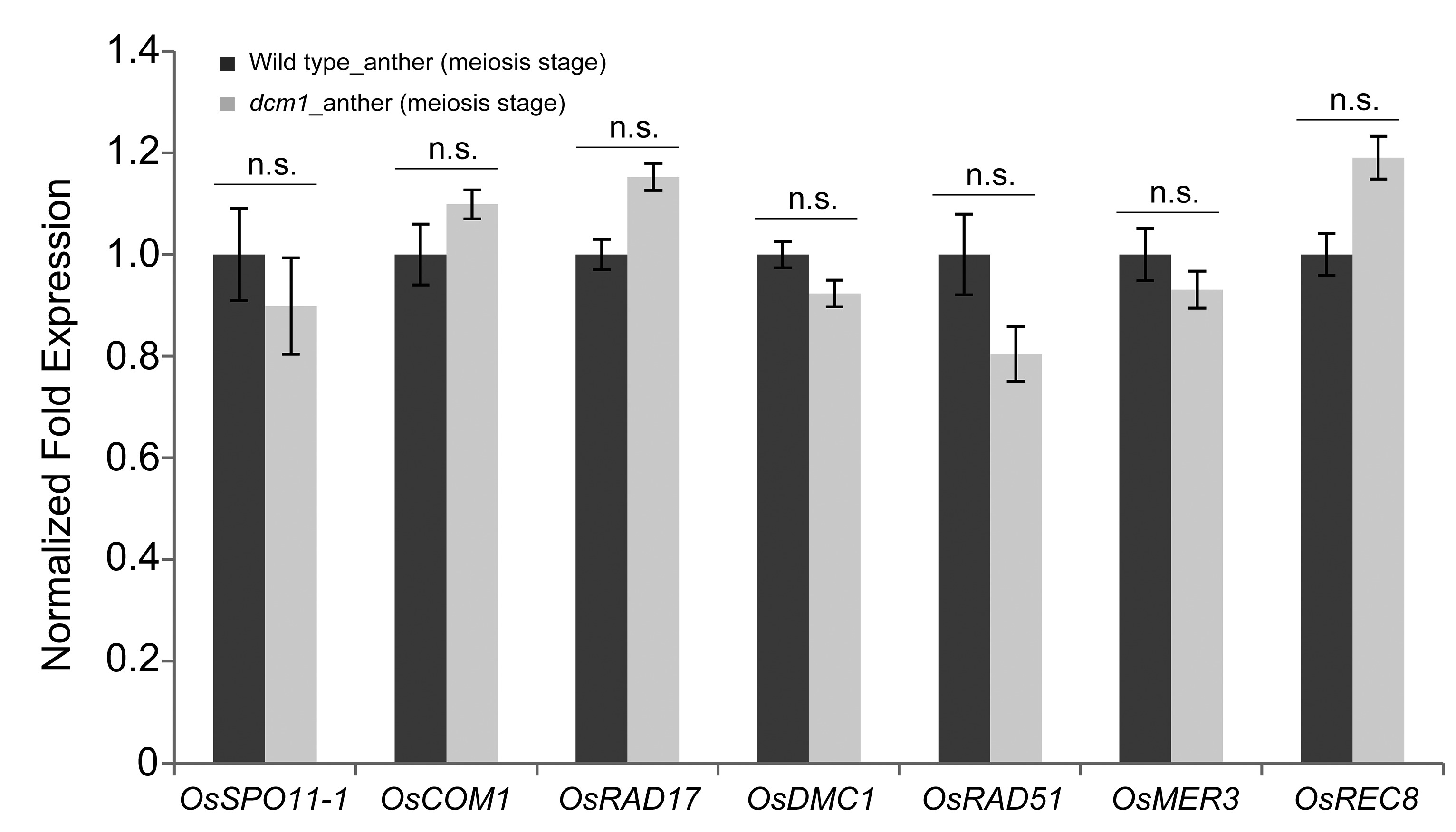

Supplement: S5 Fig — Ubiquitin is used as endogenous control. Error bars represent SD (n = 3). n.s., no significant differences, P>0.05 in two-tailed Student’s t-tests. (TIF) [file pgen.1007769.s005.tif]
