# Supplementary material for: The zinc finger protein DCM1 is required for male meiotic cytokinesis by preserving callose in rice
Source: PLoS Genet. 2018 Nov 12;14(11):e1007769. doi: 10.1371/journal.pgen.1007769 (PMC6258382; doi:10.1371/journal.pgen.1007769)
Supplement: S1 Table — (DOCX) [file pgen.1007769.s006.docx]

|  | Spikelets for hybridization | Number of seeds | seed setting rate | *p* value |
| --- | --- | --- | --- | --- |
| Wild type | 52 | 41 | 78.8% |  |
|  | 26 | 20 | 76.9% |  |
|  | 47 | 34 | 72.3% |  |
| *dcm1* | 49 | 40 | 81.6% | 0.6498 |
|  | 38 | 28 | 73.7% |  |
|  | 48 | 37 | 77.1% |  |
