# Supplementary material for: The zinc finger protein DCM1 is required for male meiotic cytokinesis by preserving callose in rice
Source: PLoS Genet. 2018 Nov 12;14(11):e1007769. doi: 10.1371/journal.pgen.1007769 (PMC6258382; doi:10.1371/journal.pgen.1007769)
Supplement: S2 Table — (DOCX) [file pgen.1007769.s007.docx]

| **Mapping** | **C6-25.22F(AP004737-30)** | **GCATCTATCTTTCTCCCTT** |
| --- | --- | --- |
|  | **C6-25.22R(AP004737-30)** | **CTGTAGCCCTCATAAGCA** |
|  | **C6-26.28F(AP003568-57)** | **TTCCCATGAACTAATTCTAACC** |
|  | **C6-26.28R(AP003568-57)** | **TCCCTCCAACCAAACACTA** |
|  | **C6-25.7F(AP004792-106)** | **GGCATAGGAGGCAGAGTG** |
|  | **C6-25.7R(AP004792-106)** | **TAATGGCAGCAAAGCAAA** |
|  | **R3879-F** | **CACTAATCAAGCCACTTCGG** |
|  | **R3879-R** | **CGAAACTTGTTTTCCTTCCC** |
|  | **C6-25.86F(AP005386-146)** | **TGATTTGCCTACTCTGCT** |
|  | **C6-25.86R(AP005386-146)** | **ATTGTAAGGAACCGTGCT** |
|  | **C6-26.003F(AP003571-34)** | **GGATTTACTGCCGTGCTT** |
|  | **C6-26.003R(AP003571-34)** | **TTGTTCTTGATCCCACCA** |
| **CRISPR-CAS9** | ***DCM1*-CAS9-F** | **ggca****TTGGATTTGACTTTGCTCTT** |
|  | ***DCM1*-CAS9-R** | **aaacAAGAGCAAAGTCAAATCCAA** |
| **Full-length cDNA cloning** | **CDS-F** | **ATGGATCCGCCTCCCCCGTT** |
|  | **CDS-R** | **TCAAACTCTTGCTGTTCTCA** |
|  | ***DCM1*-3'RACE-OUTER** | **GGATGATGATGCGGATGAAG** |
|  | ***DCM1*-3'RACE-INNER** | **GCACTAATCAAGCCACTTCG** |
|  | ***DCM1*-5'RACE-OUTER** | **CCACGGGAGAAGCTGTTGCA** |
|  | ***DCM1*-5'RACE-INNER** | **CCCGTGGCGATGGTAGTGGT** |
| **RT-PCR** | ***DCM1*-RT-F7** | **ACTGACTCACAAGGTCCTTC** |
|  | ***DCM1*-RT-R7** | **CTGTGCTTTTTATGACACTC** |
|  | **OsGSL3-Qrt1f** | **GTGGCACGTTTGTATGAGAT** |
|  | **OsGSL3-Qrt1r** | **CTAACTGCGATAACCTTGTT** |
|  | **OsGSL2-Qrt1f** | **ATTGTTATGGCACCAGTAGC** |
|  | **OsGSL2-Qrt1r** | **GGAAGCCTGTATGAACCTTG** |
|  | **CRR1-F** | **GTCATTGCCAAGTTAGTTGCTGTAGCAG** |
|  | **CRR1-R** | **GCCACTCATATCAACAGACAGCACTTC** |
|  | **OsGSL1-Qrt1f** | **CAACTCCGAAGAAGTCAACC** |
|  | **OsGSL1-Qrt1r** | **TACATCCGAGCAATCTCACG** |
|  | **OsGSL4-Qrt1f** | **TTGGCATTCATCCCTACTGG** |
|  | **OsGSL4-Qrt1r** | **TACATCCTCGCCATCTCCTG** |
|  | **OsGSL6-Qrt1f** | **GCCATCCCTCACATGACAGT** |
|  | **OsGSL6-Qrt1r** | **GACACGAAAGGAAACCAAGC** |
|  | **OsGSL7-Qrt1f** | **ATGACAGTCCAGGACATCTT** |
|  | **OsGSL7-Qrt1r** | **GATACAAACGGGAACCAAGC** |
|  | **OsGSL9-Qrt1f** | **ATTCGTCGTTTGTAACCTCA** |
|  | **OsGSL9-Qrt1r** | **ACAGCATTGCCTTCTTCACC** |
|  | **OsGSL10-Qrt1f** | **GTGCTTTCTGGCATTCTTAC** |
|  | **OsGSL10-Qrt1r** | **AGTCCTCTACTGAACGCTTG** |
|  | **GSL5-qrt-1F** | **GGTCCTGAAGGTGGTATCCA** |
|  | **GSL5-qrt-1R** | **GGCTTGCTGGCTTGAGATAT** |
|  | **OSG1-qrt-1F** | **GCGACCAAAGATAATGCTAG** |
|  | **OSG1-qrt-1R** | **TTGGGTACACAGGGCTCTTG** |
|  | **UPG1-QRT-1F** | **GTTCAAGAAGGTTGCCAATT** |
|  | **UPG1-QRT-1R** | **TCCTCCGGACCGTTGATGTC** |
|  | **abcg15-6f** | **CATGTGTGGCCAACCAAAGA** |
|  | **abcg15-6r** | **GTGGTCTTGCCACTGCCAGA** |
|  | **DPW-QRTF** | **GTAGTCGGAGATGTCAGAGAAGCC** |
|  | **DPW-QRTR** | **GATCCCTCCAGGTGCTCTCG** |
|  | **OsA6-RT-F** | **GACGTGCTACTTCAACAA** |
|  | **OsA6-RT-R** | **CCTGGGTCTAAATCTAATC** |
|  | **OsMYB80-RT-F** | **TCTCCTACATCACCCAGTAC** |
|  | **OsMYB80-RT-R** | **CTTCACGTCGTTGTCCGT** |
|  | **SPO11-RT1F** | **GAGAAGGAGACAGTGTTTCAA** |
|  | **SPO11-RT1R** | **TGCAGATAACGCCTCAATCTCA** |
|  | **COM1-RT1F** | **CTCCGGAAGCCCTCCAAGCAA** |
|  | **COM1-RT1R** | **CGAGTTACTGAATGAACTCCT** |
|  | **rad17-rt-1f** | **ATCCGTAGCCCAAGAGTT** |
|  | **rad17-rt-1r** | **TTGCGAGAACCAGATGTAC** |
|  | **DMC1-RT1F** | **GTCCAAGCAGTACGACGAAG** |
|  | **DMC1-RT1R** | **TCTCCAGAGTTTATCCCTTGC** |
|  | **rad51-rt-1f** | **AAGTTTCTGAGGAGCCTTCA** |
|  | **rad51-rt-1r** | **AAGCCTTGTTGTGGAAGCAT** |
|  | **MER3-RT1F** | **CAACTTAGCTTCACTGGAGG** |
|  | **MER3-RT1R** | **CTGGATTTCTTGCGTATGTG** |
|  | **REC8-RT1F** | **TCCACTCGTACCTCAAGCTA** |
|  | **REC8-RT1R** | **GTTGCTAAAACGCATGCTTG** |
| **Y2H** | **DCM1-BD-F** | **TGGCCATGGAGGCCGAATTCATGGATCCGCCTCCCCCGTT** |
|  | **DCM1-BD-R** | **GCTGCAGGTCGACGGATCCCTCAAACTCTTGCTGTTCTCA** |
|  | **DCM1_1-1293_-BD-R** | **GCTGCAGGTCGACGGATCCCCGGAGTGGACTTGCATGGTG** |
|  | **DCM1_1294-2068_-BD-F** | **TGGCCATGGAGGCCGAATTCCTCACTGTTGGCTTGCCAGA** |
|  | **DCM1_1294-1772_-BD-R** | **GCTGCAGGTCGACGGATCCCTTGATTGCCAGTGACAGATT** |
|  | **DCM1_1773-2068_-BD-F** | **TGGCCATGGAGGCCGAATTCTTAAGAAACAGCCGCCAATC** |
|  | **DCM1_1294-2068_-AD-F** | **CCATGGAGGCCAGTGAATTCCTCACTGTTGGCTTGCCAGA** |
|  | **DCM1-AD-R** | **GCTCGAGCTCGATGGATCCCTCAAACTCTTGCTGTTCTCA** |
|  | **DCM1_1294-1772_-AD-R** | **GCTCGAGCTCGATGGATCCCTTGATTGCCAGTGACAGATT** |
|  | **DCM1_1773-2068_-AD-F** | **CCATGGAGGCCAGTGAATTCTTAAGAAACAGCCGCCAATC** |
|  | **OsPABN1-AD-F** | **CCATGGAGGCCAGTGAATTCATGGACGAGGAGGAGCACGA** |
|  | **OsPABN1-AD-R** | **GCTCGAGCTCGATGGATCCCTCAAAAGTATGGCCTGTAGC** |
|  | **OsPABN1-BD-F** | **TGGCCATGGAGGCCGAATTCATGGACGAGGAGGAGCACGA** |
|  | **OsPABN1-BD-R** | **GCTGCAGGTCGACGGATCCCTCAAAAGTATGGCCTGTAGC** |
|  | **OsPABN2-AD-F** | **CCATGGAGGCCAGTGAATTCATGGCGGACGAGGAGCACGA** |
|  | **OsPABN2-AD-R** | **GCTCGAGCTCGATGGATCCCTCAAAAGTATGGCCTGCGTG** |
|  | **OsPABN2-BD-F** | **TGGCCATGGAGGCCGAATTCATGGCGGACGAGGAGCACGA** |
|  | **OsPABN2-BD-R** | **GCTGCAGGTCGACGGATCCCTCAAAAGTATGGCCTGCGTG** |
| **BiFC** | **pVYNER-DCM1-F** | **CCCAGGCCTACTAGTGGATCCATGGATCCGCCTCCCCCGTT** |
|  | **pVYNER-DCM1-2R** | **CCCGGGAGCGGTACCCTCGAGTCAAACTCTTGCTGTTCTCA** |
|  | **pVYNER-PAB2-1-F** | **CCCAGGCCTACTAGTGGATCCATGGACGAGGAGGAGCACGA** |
|  | **pVYNER-PAB2-1-R** | **CCCGGGAGCGGTACCCTCGAGTCAAAAGTATGGCCTGTAGC** |
|  | **pVYNER-PAB2-2-F** | **CCCAGGCCTACTAGTGGATCCATGGCGGACGAGGAGCACGA** |
|  | **pVYNER-PAB2-2-R** | **CCCGGGAGCGGTACCCTCGAGTCAAAAGTATGGCCTGCGTG** |
| **ISH** | **DCM1-ISH-F** | **TGTCTCAACTGATGGGTTCT** |
|  | **DCM1-ISH-R** | **CATGCGGATTACATTCTTCG** |
